# A Randomized Trial Comparing the Diagnostic Accuracy of Visual Inspection with Acetic Acid to Visual Inspection with Lugol’s Iodine for Cervical Cancer Screening in HIV-Infected Women

**DOI:** 10.1371/journal.pone.0118568

**Published:** 2015-04-07

**Authors:** Megan J. Huchko, Jennifer Sneden, Jennifer M. Zakaras, Karen Smith-McCune, George Sawaya, May Maloba, Elizabeth Ann Bukusi, Craig R. Cohen

**Affiliations:** 1 Department of Obstetrics, Gynecology and Reproductive Sciences, University of California San Francisco, San Francisco, California, United States of America; 2 Department of Epidemiology and Biostatistics, University of California San Francisco, San Francisco, California, United States of America; 3 Family AIDS Care and Education Services, Kenya Medical Research Institute, Kisumu, Kenya; 4 Center for Microbiology Research, Kenya Medical Research Institute, Nairobi, Kenya; 5 Department of Obstetrics and Gynecology, University of Nairobi, Nairobi, Kenya; Rush University, UNITED STATES

## Abstract

Visual inspection with Acetic Acid (VIA) and Visual Inspection with Lugol’s Iodine (VILI) are increasingly recommended in various cervical cancer screening protocols in low-resource settings. Although VIA is more widely used, VILI has been advocated as an easier and more specific screening test. VILI has not been well-validated as a stand-alone screening test, compared to VIA or validated for use in HIV-infected women. We carried out a randomized clinical trial to compare the diagnostic accuracy of VIA and VILI among HIV-infected women. Women attending the Family AIDS Care and Education Services (FACES) clinic in western Kenya were enrolled and randomized to undergo either VIA or VILI with colposcopy. Lesions suspicious for cervical intraepithelial neoplasia 2 or greater (CIN2+) were biopsied. Between October 2011 and June 2012, 654 were randomized to undergo VIA or VILI. The test positivity rates were 26.2% for VIA and 30.6% for VILI (p = 0.22). The rate of detection of CIN2+ was 7.7% in the VIA arm and 11.5% in the VILI arm (p = 0.10). There was no significant difference in the diagnostic performance of VIA and VILI for the detection of CIN2+. Sensitivity and specificity were 84.0% and 78.6%, respectively, for VIA and 84.2% and 76.4% for VILI. The positive and negative predictive values were 24.7% and 98.3% for VIA, and 31.7% and 97.4% for VILI. Among women with CD4+ count < 350, VILI had a significantly decreased specificity (66.2%) compared to VIA in the same group (83.9%, p = 0.02) and compared to VILI performed among women with CD4+ count ≥ 350 (79.7%, p = 0.02). VIA and VILI had similar diagnostic accuracy and rates of CIN2+ detection among HIV-infected women.

**Trial Registration:**

ClinicalTrials.gov NCT02237326

## Introduction

Cervical cancer is the fourth most common cancer in women, with an estimated 527,624 cases diagnosed globally in 2012.[1] Nearly 85% of cases and 87% of deaths occur in less developed regions, particularly in sub-Saharan Africa where several factors conspire to make cervical cancer a leading cause of cancer and cancer-related mortality.[2] Inadequate health care and public health infrastructure, competing health priorities, and persistent poverty prevent large-scale cervical cancer prevention programs from gaining traction; today, only an estimated 5% of women in Sub-Saharan Africa have ever been screened.[3] High rates of HIV infection in the region further escalate cervical cancer incidence through an increased risk for human papillomavirus (HPV) infection—the primary cause of cervical cancers and precancerous cervical lesions—and an accelerated incidence and progression of cervical neoplasia.[4] Growing anti-retroviral use in recent years is extending lifespans for HIV-infected women, without a clear benefit for cervical cancer outcomes. This increases the number of women living longer with excess cervical cancer risk,[5] making the implementation of effective screening programs an urgent public health priority these low-resource settings.

Cytology-based screening strategies have been effective in reducing rates of cervical cancer in the United States and other developed countries but are infeasible in low-resource settings. The need for skilled personnel, laboratory equipment, and the transport and storing of cervical specimens are key barriers for many rural areas in countries facing considerable infrastructure and cost constraints. Referrals for follow-up and treatment can also lead to high rates of attrition, highlighting the importance of same day results.[6] To avoid such bottlenecks, the World Health Organization (WHO) recommends a “screen-and-treat” program in low-resource settings that specifies treatment, often with cryotherapy, soon or immediately after a positive screening test without confirmatory diagnosis.[7] Absent a diagnostic procedure, the screening methods should detect as many cervical cancer and precancer cases as possible while also avoiding excessive adverse events from overtreatment. While studies in low-resource settings have shown that HPV testing is most effective in reducing cervical intraepithelial neoplasia grade 2 or worse lesions, including cervical cancer,[8] at present, costs can be prohibitive and the reality remains that most countries do not have the resources to implement widespread HPV testing.

Visual inspection with acetic acid (VIA) and visual inspection with Lugol’s iodine (VILI) are low-cost screening technologies that have been implemented as alternatives to cytology-based programs, with VIA being the most widely studied and used. By highlighting precancerous lesions in a way that can be seen by the naked eye, visual inspection techniques can be administered in clinics by trained non-physician clinicians with immediate results. Although there are some drawbacks to the use of visual screening methods, including the tests’ subjectivity which leads to varying accuracy in different settings, numerous studies have found VIA to have acceptable sensitivity and specificity[9] and cost-effectiveness[10] for use as a screening tool in low-resource settings. Some authors have recommended adjunctive testing with VIA followed by VILI (VIA/VILI) to improve test performance[11] or the use of VILI alone.[12] [13] While both VIA and VILI have been found to have similar or higher sensitivity compared to conventional cytology,[14] VIA has lower specificity than VILI[15] and decreased accuracy in the presence of infection, such as cervicitis.[16] The subjective nature of visual screening makes test performance dependent on the training and understanding of the clinician performing the test, and some studies have found that healthcare providers find it easier to interpret the color-patterns produced by VILI’s iodine staining, rather than VIA’s acetic acid.[17,18] While this suggests that VILI may be a more straightforward test, Lugol’s iodine is more difficult to procure than acetic acid, exacerbating existing supply challenges facing resource-limited settings, and clinicians must wait up to an hour for the iodine stain to fade before administering an alternative test, such as colposcopy or VIA as a second test.

With a number of developing countries introducing visual inspection screening methods on a national or pilot basis,[19] it is critical to ensure the most effective, feasible, and sustainable strategies are pursued. VIA has been well-studied in low-resource populations and among HIV-infected women as a standalone screening test. VILI, on the other hand, is far less studied as a primary test and is usually evaluated in conjunction with a second screening method, such as VIA, when it is administered after the application of acetic acid.[20] VIA and VILI have been shown to perform similarly in low-resource settings, [21,22] though to our knowledge this has not been explored in an HIV-infected cohort. Studies have suggested that HIV-related factors, such as increased inflammation, may affect the sensitivity and specificity of visual screening methods among women with HIV.[23] To inform cervical cancer screening guidelines about whether VILI should be used alone or in conjunction with a second screening technology, we sought to examine and compare the test characteristics of VIA and VILI as stand alone screening tests among HIV-infected women in an HIV-primary care setting in western Kenya.

## Methods

We conducted a randomized clinical trial to compare the sensitivity, specificity, and positive and negative predictive values of VIA and VILI for the detection of cervical intraepithelial neoplasia 2 or worse (CIN2+). Recruitment and enrollment took place between October 2011 and June 2012. Women attending the Family AIDS Care and Education Services (FACES) clinics in Kisumu, Kenya were consecutively recruited to participate if they were eligible for cervical cancer screening per the Kenya Ministry of Health guidelines (age 23–65, intact uterus and cervix) and had not been previously diagnosed with or treated for CIN2+. After providing informed consent, women were randomized to undergo either VIA or VILI in a 1:1 allocation. Randomization was done at a central site by the data manager in blocks of five through the computer program Stata 11 (StataCorp, College Station, TX). Randomization assignment was given after enrollment by the study coordinator. Regardless of randomization arm, women underwent a pelvic inspection with naked visual inspection of the cervix to confirm eligibility. Women with evidence of cervicitis (mucopurulent discharge or friability) were considered ineligible and offered treatment per the clinic’s guidelines for syndromic management of cervicitis.[24]

Cervical exams were performed by three study staff (two nurses and one clinical officer) who had been trained and certified to perform VIA, VILI and colposcopy and had each performed over 1200 VIAs, 400 VILIs and 400 colposcopies prior to study initiation. Women in the VIA arm had a naked eye evaluation of the cervix to identify landmarks, followed by application of 5% acetic acid and a re-evaluation of the squamocolumnar junction (SCJ) after one to two minutes, using a standard exam lamp as a light source. VIA was considered satisfactory if the entire SCJ was identified. The appearance of well-defined or raised dense aceto-white lesions near the SCJ indicated a positive result. This was immediately followed by a colposcopy, performed by a second provider, blinded to the VIA results. Colposcopic exam included naked eye inspection, re-application of acetic acid, green filter and Lugol’s iodine. A cotton swab was used to assist clinicians in visualizing the endocervical canal if the entire SCJ was not seen. Colposcopy results were reported as normal, inflammation, probably CIN1, probably CIN2+, or suspicious for cancer. Biopsies were based on colposcopic findings, regardless of VIA or VILI result. Women underwent colposcopically-directed biopsy for any unsatisfactory colposcopies or for lesions suspicious for CIN2+ or cancer. No biopsies were performed in women with normal colposcopic exams; these women were determined to have “no disease” based on colposcopic findings. Due to interference in the colposcopic exam from the staining properties of Lugol’s iodine, women in the VILI arm underwent colposcopic exam first, followed by VILI performed by a second, blinded provider. A VILI was considered to be positive if the clinician identified any mustard-yellow non-iodine staining flat or raised lesions near the SCJ. After VILI was completed, the clinician performing the colposcopy returned to the room in order to evaluate the cervix with Lugol’s iodine and make the final decision to perform a biopsy.

Biopsy specimens were stored in 10% buffered formalin at room temperature in the FACES lab for up to week, then sent in batches to the Department of Human Pathology anatomical pathology lab at the University of Nairobi for interpretation. Specimens were read separately by two histopathologists who were blinded to the results of the VIA. Results were reported using the WHO classification system for gynecologic tumors.[25] For specimens with more than one diagnosis, the outcome was defined as the most severe diagnosis. Final diagnosis for discrepant results from the same specimen was determined by consensus between the two pathologists. Women with CIN2+ confirmed on biopsy were offered treatment with Loop Electrosurgical Excision Procedure (LEEP) in the clinic. Women with invasive cancer were offered LEEP in the clinic (Stage IA1) or referral to the provincial hospital for further staging and surgical or medical management.

Baseline demographic and clinical data was collected at the time of the visit, including age, marital status, education, reproductive history, last menstrual period, contraceptive use and current HAART regimen. The three-drug HAART regimens were those available at FACES, per the Kenya Ministry of Health Guidelines. First-line nucleoside reverse transcriptase inhibitor- (NRTI) based regimens contained zidovudine, stavudine or tenofivir plus lamivudine plus a non-nucleoside reverse transcriptase inhibitor (NNRTI), either nevirapine or efavirenz. Second-line NRTI-based regimens included lopinavir/ritonavir, plus lamivudine or abacavir and an NRTI. Additional clinical variables were obtained from the paper file (reviewed at the time of the study visit) and the electronic medical record. These included verification of current and previous HAART regimens, WHO Stage, all documented CD4+ counts, time since HIV-diagnosis and duration of enrollment into HIV-care. CD4+ nadir has been shown to be an effective indicator of immune status as it relates to risk for cervical disease, so we defined the lowest CD4+ count measured while in care as a proxy for CD4+ nadir.

For test performance calculations for the primary outcome, VIA or VILI results were categorized as negative or positive. Colposcopy results were categorized as presence or absence of biopsy-confirmed CIN2+. We based our sample size calculations on the CIN2+ prevalence of 7% in this clinic.[26] Assuming a sensitivity and specificity reached at least 65%, to determine a difference of at least 10% in test characteristics between the two screening tests, with a two-sided alpha of 0.05, we needed to enroll 325 women in each arm. This sample size will provide power to detect smaller differences if the sensitivity and specificity were found to be higher. We performed a sensitivity analysis for each test characteristic using CIN1+ as the outcome. In order to examine potential confounding by known and postulated clinical and demographic characteristics, we performed a pre-specified secondary analysis of test performance stratified by age, CD4+ count and HAART status. The stratified analysis looked at differences within and between the randomization arms. To compare demographic and clinical characteristic between randomization arms, a Pearson’s chi-squared test for categorical variables while the Student’s t-test was used for continuous variables. We used bivariate logistic regression to detect test characteristic differences within and between strata. Data cleaning and statistical analysis were performed using Stata 12 (StataCorp, College Station, TX, See S1 Dataset).

This study was approved by the ethical review boards at the University of California, San Francisco (Committee for Human Subjects Research, #10–00197) and the Kenya Medical Research Institute (Ethical Review Committee, #1825). This study is registered as a clinical trial (Clinicaltrials.gov, NCT:02237326, See S1 Protocol and S1 CONSORT Checklist). As all participants received the reference standard for diagnosis of cervical lesions at the time of their study visit, there was no potential for differential in health outcomes by study arm. For this reason, trial registration was done post-recruitment to allow for tracking of study results. The authors confirm that all ongoing and related trials for this drug/intervention are registered. All women signed informed consent in their preferred language (English, Kiswahili or Dholuo) prior to participation.

## Results

**Fig 1:**
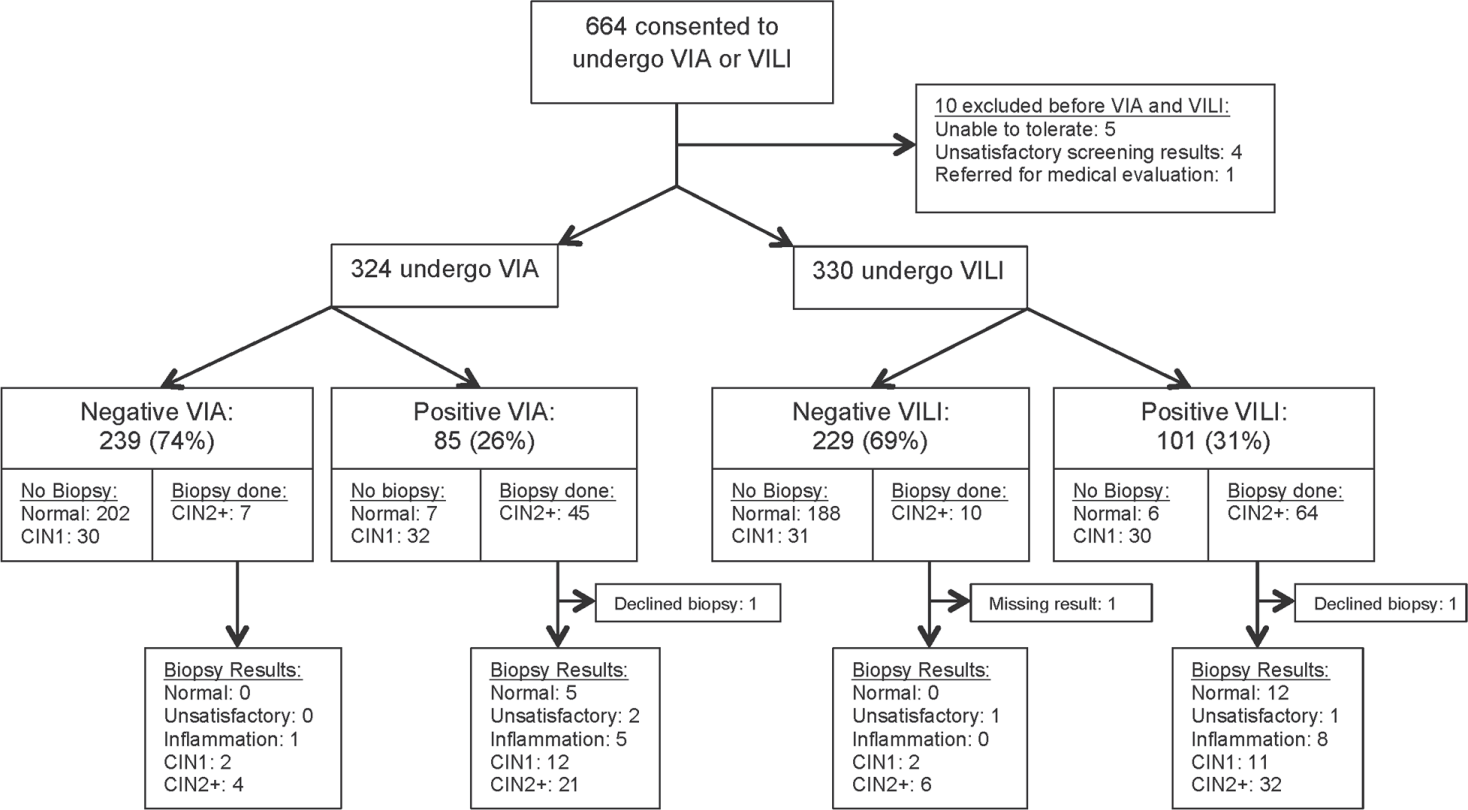
Flowsheet of enrollment, randomization and screening results for VIA and VILI.

During the eight-month recruitment period, 664 women were recruited for the study. 654 were eligible for randomization and underwent VIA (N = 324) or VILI (N = 330). Reasons for exclusion are shown in Fig. 1. The average participant age was 34.5 years (SD = 8.1), 342 women (52.7%) were married and 476 (72.8%) reported one current partner. Women had delivered an average of three children, and 257 (39.3%) were on some form of contraception at the time of screening. Over half of women were WHO Stage 1 or 2 (57.5%, N = 376). The mean CD4+ count at screening was 544 cells/dl (SD = 257) and 500 women (76.5%) were on HAART. There were no significant differences in any of the clinical or demographic characteristics between the two randomization arms (Table 1). There were no adverse events related to the screening test in either the VIA or VILI arm.

**Table 1:** Baseline demographic and clinical characteristics of HIV-infected women randomized to cervical cancer screening with Visual Inspection with Acetic Acid (VIA) or Visual Inspection with Lugol's Iodine (VILI).

Characteristic	VIA	VILI	p-value
	*(n* = 324)	*(n* = 330)	
Age (years), mean, (SD)	34.4 (8.2)	34.6 (8.1)	0.81
**Relationship Status, no., (%)** ^**a**^			
Single	19 (5.9%)	19 (5.8%)	
Married	174 (54.4%)	168 (51.1%)	
Divorced	33 (10.3%)	43 (13.1%)	
Widowed	94 (29.4%)	99 (30.1%)	0.32
**Number of current partners,** ^**b**^ **no., (%)**			
0	79 (24.4%)	89 (27.0%)	
1	241 (74.4%)	235 (71.2%)	
>1	4 (1.2%)	6 (1.8%)	0.55
Number of lifetime partners, mean, (SD)	4.8 (3.4)	5.0 (4.1)	0.37
Employed, no., (%)^a^	317 (98.5%)	318 (97.9%)	0.57
**Reproductive History**			
Gravidity, mean, (SD)	3.6 (2.4)	3.4 (2.1)	0.37
Parity, mean, (SD)	2.9 (2.1)	2.8 (1.8)	0.25
Underwent prior c-section, no., (%)	33 (10.2%)	31 (9.4%)	0.74
Amenorrhea, no., (%)^a^	57 (17.7%)	43 (13.2%)	0.12
Menopause, no., (%)^a^	28 (8.7%)	31 (9.5%)	0.71
**Contraception Use,** ^**c**^ **no., (%)** ^**a**^	126 (39.0%)	131 (39.7%)	0.83
**Contraception use by type,** ^**d**^ **no., (%)**			
Oral contraceptives	13 (10.3%)	10 (7.6%)	
Injectable (Depo Provera™)	69 (54.8%)	75 (57.3%)	
Implant (Jadelle or Norplant)	20 (15.9%)	27 (20.6%)	
Intrauterine Device in-situ	3 (2.4%)	5 (3.8%)	
Tubal Ligation	21 (16.8%)	14 (10.7%)	0.99
**HIV-related characteristics**			
Time since first HIV-diagnosis, mo., (SD)	44.6 (28.1)	45.5 (27.0)	0.67
Most advanced WHO clinical stage, no., (%)			
1	93 (28.7%)	98 (29.7%)	
2	96 (29.6%)	89 (27.0%)	
3	115 (35.5%)	113 (34.2%)	
4	20 (6.2%)	30 (9.1%)	0.69
CD4+ at screening visit, mean, (SD)	559.4 (261.3)	529.1 (252.7)	0.13
CD4+ nadir, mean cells/dL, no., (%)			
<200	26 (8.0%)	27 (8.2%)	
201–349	36 (11.1%)	53 (16.0%)	
350–500	75 (23.2%)	78 (23.6%)	
>500	187 (57.7%)	172 (51.1%)	0.12
On HAART, no., (%)^a^	246 (75.9%)	254 (76.7%)	0.75
Duration on HAART, mo., (SD)	22.1 (20.8)	21.2 (19.4)	0.63
On HARRT > = 6 months, no., (%)	180 (73.2%)	182 (71.7%)	0.70
On first-line HAART regimen,^e^ no., (%)	224 (91.1%)	230 (90.6%)	0.845

SD, standard deviation; WHO, World Health Organization; CD4+ nadir, lowest measured CD4+ count while in care; HAART, highly active retroviral therapy.

^a^ Variable with missing data. Columns represent the % among complete answers.

^b^ Current partner defined as living with a spouse or unmarried partner.

^c^ Excluding condom use.

^d^ Represents the use of one contraceptive method at a time.

^e^ First-line HAART regimens consisted of a triple drug combination of either zidovudine or stavudine+lamivudine+either nevirapine or efaviranz.

There was no significant difference in the performance of VIA and VILI for the detection of biopsy-confirmed CIN2+. The test positivity rates were 26.2% for VIA and 30.6% for VILI (p = 0.22). For VIA, sensitivity was 84.0% and specificity was 78.6%. For VILI, sensitivity was 84.2% and specificity was 76.4%. The positive and negative predictive values were 24.7% and 98.3% for VIA, and 31.7% and 97.4% for VILI. There was a non-significant trend toward increased detection of CIN2+ among women in the VILI arm (11.5% versus 7.7%, p = 0.10, relative risk 1.50, 95% CI 0.88–2.53). When evaluating clinically or histologically diagnosed CIN1+ as an outcome, the sensitivity of both tests decreased, and the specificity increased (Table 2).

**Table 2:** Clinical performance of VIA and VILI for diagnosis of CIN.

A. Bx Confirmed CIN 2+^a^					
	VIA		VILI		p-value
	%	95% CI	%	95% CI	
Sensitivity	84.0%	64.0%–95.5%	84.2%	68.7%–94%	0.98
Specificity	78.6%	73.5%–83.1%	76.4%	71.2%–81.3%	0.52
PPV	24.7%	16.0%–35.3%	31.7%	22.8%–41.7%	0.30
NPV	98.3%	95.8%–99.5%	97.4%	94.4%–99.0%	0.48
Test Positivity Rate	26.2%	21.4%–31.1%	30.6%	25.6%–35.6%	0.22
Prevalence	7.7%	5.1%–11.2%	11.5%	8.3%–15.5%	0.10

B. CIN1+^b^					
	VIA		VILI		p-value
	%	95% CI	%	95% CI	
Sensitivity	64.4%	52.2%–73.6%	65.2%	55.6%–73.9%	0.90
Specificity	91.0%	86.5%–94.4%	87.2%	82.0%–91.3%	0.19
PPV	76.5%	66.0%–85.0%	72.3%	62.5%–80.7%	0.52
NPV	84.9%	79.8%–89.2%	83.0%	77.5%–87.6%	0.56
Test Positivity Rate	26.2%	21.4%–31.1%	30.6%	25.6%–35.6%	0.22
Prevalence	31.2%	26.0%–36.5%	33.9%	29.0%–39.3%	0.45

2x2 Tables for Each Analysis					
A. Bx Confirmed CIN2+					
VIA	CIN2+	Not CIN2+	VILI	CIN2+	Not CIN2+
Positive	21	64	Positive	32	69
Negative	4	235	Negative	6	223
B. CIN1+					
VIA	CIN1+	Not CIN1+	VILI	CIN1+	Not CIN1+
Positive	65	20	Positive	73	28
Negative	36	203	Negative	39	190

CIN2+, cervical intraepithelial neoplasia 2 or greater.

^a^ Analyses exclude two missing screening results.

^b^ Diagnosis of CIN2+ confirmed by biopsy; CIN1 diagnosis made by colposcopic impression or biopsy.

When stratifying the cohort by age, CD4+ count, and HAART status, there were several differences on test performance between tests within clinical strata and between strata for a specified test (Table 3). When comparing test characteristics of VIA and VILI within younger or older age groups, there was no difference between the two tests. However, compared to younger women, VILI had decreased test positivity and biopsy-confirmed CIN2+ detection in older women, with a trend toward increased specificity. Among women with a CD4+ < 350, VILI had a higher test positivity rate and a lower specificity compared to VIA. When comparing women with higher CD4+ counts to those < 350, VILI specificity and test positivity increased.

**Table 3:** Clinical performance of VIA or VILI for detection of CIN2+, stratified by age, CD4+ count, and HAART use.

	Age < = 35					Age >35					Within test, between strata^a^	
	VIA (*n* = 199)	95% CI	VILI (*n* = 200)	95% CI	p-value	VIA (*n* = 125)	95% CI	VILI (*n* = 130)	95% CI	p-value	VIA	VILI
Sensitivity	84.2%	60.4%–96.6%	86.2%	68.3%–96.1%	0.85	83.3%	35.9%–99.6%	77.8%	40%–97.2%	0.79	0.96	0.55
Specificitiy	76.1%	69.2%–82.1%	72.5%	65.2%–79.1%	0.44	82.4%	74.3%–88.7%	81.8%	73.8%–88.2%	0.91	0.20	0.07
PPV	27.1%	16.4%–40.3%	34.7%	23.9%–46.9%	0.35	19.2%	6.6%–39.4%	24.1%	10.3%–43.5%	0.66	0.44	0.30
NPV	98.0%	93.9%–99.6%	97.9%	92.2%–99.1%	0.99	98.4%	94.5%–100%	97.2%	93.1%–99.8%	0.43	0.51	0.59
Prevalence	9.6%	5.8%–14.5%	14.5%	9.9%–20.2%	0.13	4.8%	1.8%–10.2%	6.9%	3.2%–12.7%	0.47	0.13	**0.04**
Test Positivity	29.7%	23.2%–36.0%	36.0%	29.2%–42.7%	0.18	20.8%	13.6%–28.0%	22.3%	15.1%–29.6%	0.77	0.08	**0.01**

	CD4+ <350					CD4+ ≥350					Within test, between strata^a^	
	VIA (*n* = 62)	95% CI	VILI (*n* = 81)	95% CI	p-value	VIA (*n* = 262)	95% CI	VILI (*n* = 249)	95% CI	p-value	VIA	VILI
Sensitivity	83.3%	35.9%–99.6%	90.0%	55.5%–99.7%	0.70	84.2%	60.4–96.6%	82.1%	63.1%–93.9%	0.85	0.96	0.56
Specificitiy	**83.9%**	**71.7%–92.4%**	**66.2%**	**53.4%–76.7%**	**0.02**	77.4%	71.6%–82.5%	79.7%	73.6%–84.7%	0.54	0.28	**0.02**
PPV	35.7%	12.8%–64.9%	27.3%	13.3%–45.5%	0.56	22.5%	13.5%–34.0%	33.8%	22.8%–46.3%	0.14	0.30	0.51
NPV	97.9%	88.9%–99.9%	98.0%	88.9%–99.9%	0.99	98.4%	95.5%–99.7%	97.3%	93.7%–99.1%	0.44	0.81	0.79
Prevalence	9.7%	3.6%–19.9%	12.5%	6.2%–21.8%	0.60	7.3%	4.4%–11.1%	11.2%	7.6%–15.8%	0.12	0.52	0.79
Test Positivity	**22.6%**	**11.9%–33.3%**	**41.3%**	**30.2%–52.3%**	**0.02**	27.1%	21.7%–32.5%	27.2%	21.6%–32.8%	0.98	0.47	**0.02**

	On HAART					Not on HAART					Within test, between strata^a^	
	VIA (*n* = 246)	95% CI	VILI (*n* = 254)	95% CI	p-value	VIA (*n* = 78)	95% CI	VILI (*n* = 76)	95% CI	p-value	VIA	VILI
Sensitivity	87.5%	61.7%–98.4%	79.2%	57.8%–92.9%	0.49	77.8%	40.0%–97.2%	92.9%	66.1%–99.8%	0.30	0.53	0.29
Specificitiy	80.4%	74.7%–85.4%	78.3%	72.4%–83.4%	0.56	72.5%	60.4%–82.5%	69.3%	56.3%–80.4%	0.70	0.16	0.15
PPV	23.7%	13.6%–36.6%	27.5%	17.5%–39.6%	0.62	26.9%	11.6%–47.8%	40.6%	23.7%–59.4%	0.27	0.75	0.19
NPV	98.9%	96.2%–99.9%	97.3%	93.8%–99.1%	0.24	96.1%	86.8%–99.5%	97.7%	88.0%–99.9%	0.65	0.20	0.87
Prevalence	6.5%	3.8%–10.3%	9.5%	6.1%–13.7%	0.22	11.5%	5.4%–20.8%	18.4%	10.0%–29.0%	0.23	0.15	**0.03**
Test Positivity	24.0%	18.6%–29.4%	27.2%	21.6%–32.7%	0.41	33.3%	22.6%–44.0%	42.1%	30.7%–53.5%	0.26	0.10	**0.01**

^a^ p-value for comparison of individual screening test performance between the two strata.

## Discussion

This large, clinic-based study provides well-validated estimates of the sensitivity and specificity of VIA and VILI among HIV-infected women. In this randomized trial of 654 women, VIA and VILI performed similarly as screening tests for biopsy confimed CIN2+ among HIV-infected women. Sensitivity and specificity were similar to that seen in general populations for both cytology and VIA, although somewhat lower in this study for VILI.[12,17,27] Overall, the test positivity rates and positive predictive values were higher in our study than in those reported by others, reflective of the higher prevalence of disease in an HIV-infected population. The test positivity, as well as the sensitivity and specificity estimates, were within the range of similar studies of visual inspection screening methods performed among HIV-infected women. These results provide validation for the performance of either of these tests by clinical officers and nurses.[28,29,30,31]

VIA is much more widely studied, recommended and implemented than VILI. [7,32] VILI is rarely used as a standalone test, and is often used as a confirmatory test, either during the same exam or at a follow-up visit, prior to referral or treatment.[33] The rationale for this is based on VILI’s theoretical superior specificity for CIN2+ and potentially less confusion with inflammation or squamous metaplasia, which can lead to false-positive VIA and unnecessary treatment in screen-and-treat programs. The results of this study do not support either of those advantages for VILI among HIV-infected women. Also, VIA has some advantages in that 3–5% acetic acid is available almost everywhere, is inexpensive, and has reliable quality. In contrast, Lugol’s iodine is relatively more expensive and difficult to source in many low-resource settings, with unreliable quality that could potentially impact test diagnostic accuracy.

We did find some differences in the performance of VIA and VILI in this population when stratifying by different clinical or demographic characteristics. For example, in contrast to prior work, we saw a decrement in the specificity of VILI, but not VIA in the setting of more severe immunosuppression. Some of that difference may be attributable to increases in sub-clinical infection and inflammation present among women with lower CD4+ counts. We did not, however, adjust for multiple comparisons and, as secondary analyses, these findings are more hypothesis generating than practice changing. Thus, we do not feel that these differences are significant enough to warrant recommending one method over the other.

Our study had substantial power to compare the performance of VIA and VILI among HIV-infected women with relative precision and to evaluate the impact of clinical and demographic factors on test performance. Two additional strengths are the use of two separate clinicians for the screening test and confirmatory biopsy to reduce verification bias, and the use of colposcopy and biopsy instead of cytology to determine the outcome measure. While limiting the use of biopsy to only colposcopic exams suggestive of disease or a small proportion of negative exams has been an accepted method for studies validating cervical cancer screening tests, especially those done in resource-limited settings, [28,29,30] the visual nature of both the screening test and colposcopic assessment of the cervix to direct biopsies are intrinsically related, and likely result in overestimation of test sensitivity. The final estimation of sensitivity and specificity for both VIA and VILI are likely overestimated in this study because we did not perform random cervical biopsies or endocervical curettage in all women with a negative colposcopy. Although this would have increased the accuracy of our outcome measure,[34] it was our opinion that these additional procedures would have been unacceptable to many participants, to our community advisory board and to institutional review boards, with little additional yield in terms of clinical management of disease, as women are currently treated for CIN2 or CIN3 diagnosed through this method.[35] Additionally, we limited our sample to women with satisfactory VIA or VILI, in order to have a dichotomous result for our calculations. This potentially would inflate the sensitivity and specificity values; however, in our setting, less than 1% of women in this relatively young cohort had unsatisfactory exams. While these methodologic decisions affect the absolute accuracy estimates of each screening method, they should not affect the relative differences we report comparing the detection of CIN2+ between VIA and VILI since the participants were randomized. Finally, our study setting may not reflect real world scenarios, in which the accuracy of visual screening tests decreases without continued mentorship and quality assurance measures.

This study contributes to the ongoing evaluation of visual inspection methods for use in resource-limited settings, either in HIV-care and treatment clinics or in high HIV-prevalence areas. The test characteristics presented here will help with program planning, including protocol development and resource allocation. Our findings suggest that both tests performed with similar diagnostic accuracy, with only marginal influence by HIV-related co-factors or age. VIA was more consistent across different clinical or demographic strata. Our findings provide validation of screening tests for resource-limited settings and do not necessarily suggest a change in management or recommendations. In these settings where supplies, time, training, and continued mentorship are limited, it may make sense to focus efforts on a single screening method.

## Supporting Information

S1 CONSORT Checklist (DOC)

S1 Dataset Clean Dataset of Participant Demographic and Clinical Information. (DTA)

S1 Protocol Final Protocol for the VIA/VILI Randomized Clinical Trial. (DOCX)
