# Supplementary material for: A Randomized Trial Comparing the Diagnostic Accuracy of Visual Inspection with Acetic Acid to Visual Inspection with Lugol’s Iodine for Cervical Cancer Screening in HIV-Infected Women
Source: PLoS One. 2015 Apr 7;10(4):e0118568. doi: 10.1371/journal.pone.0118568 (PMC4388564; doi:10.1371/journal.pone.0118568)
Supplement: S1 Protocol — (DOCX) [file pone.0118568.s003.docx]

**Cervical Cancer Screening and Treatment in HIV-infected Women in Kisumu, Kenya: An Assessment of the Feasibility, Validity and Safety of New Techniques**

| **Name** | **Organization** | **Role on Project** |
| --- | --- | --- |
| Megan Huchko, MD, MPH | University of California, San Francisco, Department of Obstetrics, Gynecology, and Reproductive Sciences. Bixby Center for Global Reproductive Health | Principal Investigator |
| Elizabeth Bukusi, MB.ChB, M.Med, PhD, MPH | Kenya Medical Research Institute, University of Nairobi | Co-Investigator |
| Craig Cohen, MD, MPH | University of California, San Francisco, Department of Obstetrics, Gynecology, and Reproductive Sciences. Bixby Center for Global Reproductive Health | Co-Investigator |

**ABSTRACT**

Cervical cancer and HIV are intersecting epidemics that both disproportionately affect low-income women; the impact of the socioeconomic disparity and biologic synergy of these two diseases is seen dramatically in Kenya, where cervical cancer is the most common cancer killer. HIV-infected women are at increased risk for the development of cervical precancer and cancer, develop more aggressive lesions and are affected at younger ages. Although screening for and treatment of precancer has reduced the incidence of cervical cancer to close to zero in resource-rich countries, strategies are costly and not available in most resource-limited countries. Novel testing and treatment methods that can be carried out in low-resource settings are urgently needed to prevent cervical cancer in these settings, especially among HIV-infected women. Using a cross-sectional study design, we propose to test two potentially low-cost and effective cervical cancer screening methods: **visual inspection with acetic acid** (VIA) and **protein biomarkers** expressed in the presence of cervical dysplasia, neither of which have been well-studied in HIV-infected women. We will also randomize a small subset of women to undergo VIA compared to **Visual Inspection with Lugol's Iodine (VILI**) to compare the test characteristics of these two screening exams. In addition, remarkably little is known about the potential **impact of the presence, diagnosis and treatment of HPV-related dysplasia on HIV genital shedding, a major risk factor for HIV transmission**. To explore the impact of the presence, diagnosis and treatment of HPV-related cervical dysplasia on HIV-1 in genital secretions, we will compare the levels of HIV-1 RNA found in the cervicovaginal secretions of women with no evidence of cervical dysplasia to levels in women with high-grade cervical dysplasia. We will also compare the levels of HIV-1 RNA prior to and after cervical biopsy as well as up to 14 weeks after standard treatment for cervical dysplasia using loop electrosurgical excision procedure (LEEP). Results from this study will inform the prioritization and design of safe, effective and low-cost cervical cancer screening and prevention programs for HIV-infected women in resource-limited settings.

**GLOSSARY OF ABBREVIATIONS**

AUC…………………………………………………………………………….…Area Under the Curve

CIN………………………………………………………….………..Cervical Intraepithelial Neoplasia

FACES…………………………………………………....Family AIDS Care and Education Services

HR-HPV……………………………………………………………...High-Risk Human Papillomavirus

LEEP…………………………………………………………Loop Electrosurgical Excision Procedure

NAAT………………………………………………………………..Nucleic Acid Amplification Testing

ROC………………………………………………………………………..Responder Operating Curve

STI………………………………………………………………………Sexually Transmitted Infections

UCSF………………………………………………………...…University of California, San Francisco

VIA……………………………………………………………….......Visual Inspection with Acetic Acid

**BACKGROUND**

Invasive cervical cancer has become a rare disease in developed countries, largely due to the success of organized screening programs coupled with safe and effective treatment of precancerous lesions. Few resource-limited countries, however, have the funds or infrastructure for screening programs that detect and treat lesions before they progress to invasive cancer. The cytology-based screening programs which have substantially reduced the burden of cervical disease in developed countries are not feasible in resource-limited countries for several reasons.^1, 2^ They are costly, have high infrastructure requirements and necessitate multiple visits, which can lead to high rates of loss-to-follow up.^3-5^ Of the almost 500,000 new cases of cervical cancer diagnosed annually, 80% occur in developing countries.^1, 6^ In sub-Saharan Africa, women have a 2% to 4% lifetime risk of the disease.^1, 7^ Mortality from cervical cancer is much greater in resource-limited countries, because women are diagnosed at later stages and treatment options are limited. Cervical cancer carries an overall 80% five-year mortality rate for women in Sub-Saharan Africa, four times the rate of countries with organized screening programs.^8^ In Kenya, cervical cancer is the most common cancer among women, attributable largely to the lack of a national prevention program.^8, 9^ The exact incidence is unknown, but is estimated at approximately 40-200 per 100,000 women annually, with a two-to-threefold increase in HIV-positive women.^8, 10, 11^

Cervical cancer is an AIDS-defining malignancy, and HIV influences the risk for cervical cancer through multiple biologic pathways.^12^ HIV increases the incidence and persistence of infection with oncogenic human papillomavirus (HPV), the causative agent in cervical dysplasia cancer.^13-17^ HIV-infected women have a higher incidence and prevalence of cervical dysplasia and invasive cancer, a younger age of onset, present with more advanced malignancies and have lower survival rates than HIV-negative women.^11, 18-23^ Worsening HIV related immunosuppression is associated with more severe cervical lesions and disease progression.^24-26^ Recurrence of cervical lesions is much more common among HIV-infected than uninfected women,^27-30^ making follow-up essential for the prevention of invasive disease. The vast majority of women infected with HIV live in resource-limited settings with poor healthcare infrastructure, most notably in Sub-Saharan Africa. In these settings, limited access to cervical cancer screening programs puts them at even higher risk for cervical cancer related morbidity and mortality.^31^ HIV prevalence in Kenya was estimated to be between 4.9 and 7.3% at the end of 2007, with a prevalence in women aged 18-49 of 8.9%.^32, 33^ Before the widespread availability of anti-retroviral therapy, AIDS related deaths posed a much greater risk than cervical cancer. As the infrastructure for HIV treatment improves, women at highest risk for development of cervical disease are gaining access to the care and services that could facilitate cervical cancer prevention programs. HIV-clinics may represent the ideal setting for cervical cancer screening. To make an impact, screening and treatment strategies must be safe, low-cost and effective in HIV-infected women.

One strategy that has been proposed as a low-cost alternative to cytology screening is visual inspection of the cervix after application of 3-5% acetic acid (VIA). The technique is easily learned, has minimal infrastructure requirements and results are available immediately, allowing it to be coupled with same day treatment.^34, 35^ Several large trials have validated VIA as an effective screening method with the potential to decrease cervical cancer incidence and mortality.^36-38^ In HIV-negative women, VIA has been shown to have a similar sensitivity and specificity to HR-HPV testing and pap smears.^39-42^ However, unlike cytology screening, there have been no studies to evaluate the sensitivity and specificity of VIA in HIV-infected women.^43-47^ One of the potential drawbacks of using VIA in a see-and-treat program, however, is the undefined, but potentially high, false positive rate for cervical intraepithelial neoplasia 2/3 (CIN2/3), a precancerous lesion.^48^ Because this may lead to overtreatment, and potentially to increased infectivity, this needs to be defined in a rigorous study in a population of HIV-infected women.

In addition to low-cost, low-technology strategies for cervical cancer screening, rapidly increasing knowledge about the disease process that leads from persistent infection with HR-HPV to cervical dysplasia and cervical cancer has lead to the search for biomarkers that signal the dysplastic transformation of infected cells. Biomarker testing, either through immunohistochemical (IHC) staining of biopsy specimens or molecular assays could be used for screening or follow-up of at-risk women, triage of abnormal pap smear results or confirmation of histologic diagnoses. An ideal biomarker would be easy to collect and interpret, have a high sensitivity and specificity for precancerous lesions and could be processed with relatively simple technology in resource-limited settings. Availability of a biochemical assay for cervical dysplasia that could potentially be self-collected and read on-site would lower the costs, infrastructure and training needed for the collection and interpretation of cervical cytology specimens. One of the most promising new biomarkers undergoing evaluation is p16^INK4a^, a cyclin-dependent kinase inhibitor that is over-expressed in HPV-transformed cells.^49^ Studies among HIV-negative women have shown that IHC staining for p16^INK4a^ is strongly positive in virtually all invasive cervical cancers and is highly correlated with CIN2/3.^49^ In contrast, CIN1, which is thought to reflect transient HPV infection rather than precancerous changes, rarely stains positively for p16^INK4a^ on IHC.^50^ Therefore, presence of p16^INK4a^ on IHC may help further define abnormal lesions as high- or low-risk for cervical cancer, helping clinicians to determine which lesions need treatment. The addition of p16^INK4a^ IHC staining has been shown to improve inter-observer agreement for the diagnosis of CIN, and has been used in resource-limited settings to improve the reliability of the histological diagnosis, which is the gold-standard for making treatment decisions.^51^

A novel biochemical assay for p16^INK4a^ has been introduced that can measure biomarker levels directly from a swab of exfoliated cervical cells, rather than through the staining of cytology or biopsy specimens.^52^ The assay has been shown to have a high correlation for precancerous lesions, similar to IHC.^53^ A scoring system for the presence of p16^INK4a^ positive cells in immunocytochemistry (ICC) and biopsy specimens has been developed that increases the sensitivity (95-100%) and specificity (82-84%) for high-grade lesions.^54^ A protein-based assay with a dichotomous result for high-grade lesions has the potential to serve as a point-of-care test, which could be used as a stand-alone screening test or as an adjunct to increase the specificity of positive VIA. Although p16^INK4a^ has shown promise among HIV-negative women in resource-rich settings, to our knowledge there have been no studies testing the use of the biochemical assay among HIV-infected women or women receiving care in resource-limited settings. With this study, we seek to establish the feasibility of p16^INK4a^ biochemical assay in an HIV-clinic in a resource-limited setting, and to characterize the performance of a p16^INK4a^ biochemical assay among HIV-infected women. In addition, we will compare the test characteristics of p16^INK4a^ with the only other biomarker that is currently feasible as a point-of-care test, high-risk HPV (HR-HPV). We anticipate that this will be the first step towards the introduction of an assay that can be used as a stand-alone point-of-care screening test for cervical cancer for an extremely high-risk and vulnerable population: HIV-infected women in resource-limited settings.

Another important factor to consider in prioritizing the diagnosis of cervical dysplasia and cervical cancer in HIV-infected women is the potential for increased infectivity related to presence, diagnosis and treatment of cervical disease. This is especially important in countries such as Kenya, where HIV-prevention strategies remain paramount as healthcare resources and access to anti-retroviral therapy remain limited and the majority of infected individuals are in relationships with HIV-negative partners.^33, 55^ Understanding factors impacting HIV-transmission risk is essential to developing and implementing effective prevention strategies. The presence of HIV-1 in the genital tract plays a large role in the heterosexual and maternal-to-child transmission of HIV. ^56, 57^ The association between sexually transmitted infections (STIs) and increased risk of HIV transmission has been well studied and is thought to be partly attributable to increased levels of HIV-1 found in secretions of co-infected individuals.^58-60^ Herpes simplex virus type-2 co-infection is associated with the greatest increase in cervicovaginal HIV-1 shedding, even in the absence of genital ulcers.^61^ Increased shedding has also been seen with bacterial vaginosis,^62, 63^ *Chlamydia trachomatis*,^64^ *Neisseria gonorrhea*,^65^ *Candida* and nonspecific vaginitis.^66, 67^ Despite the high prevalence of HR-HPV, cervical dysplasia and cancer in this population, remarkably little is known about the relationship between HR-HPV-related lesions, treatment of these lesions and HIV-1 genital shedding. One prospective study among 124 HIV-infected women showed an up to 4-fold risk of HIV-1 RNA in the genital tract among women with HR-HPV, with no significant relationship to CIN. Limitations to this study include a high prevalence (50%) of cervical and vaginal infections and no participants on anti-retroviral therapy. A later study by the same investigators showed a two-fold increased risk of HIV-1 shedding among women with CIN, after controlling for plasma levels of HIV, CD4+ count and vaginal infections.^68^ A better understanding of the impact of CIN on HIV-1 genital shedding may provide additional rational for implementing screening programs in populations at high-risk for both cervical cancer and HIV transmission.

In addition, little is known about the level of HIV-1 shedding in cervix after biopsy and treatment for dysplasia. When planning for safe and effective cervical cancer prevention strategies, clinicians and investigators should consider the potential impact of CIN diagnosis and treatment on HIV-1 genital shedding. Studies have shown that the presence of inflammatory markers and cytokines in the cervix is associated with increased HIV-1 in cervicovaginal secretions,^69, 70^ although little is known about the relevance of these findings to the post-biopsy and post-LEEP cervix. Post-treatment cytology specimens have decreased specificity due to obscuring inflammatory markers related to thermal damage and repair for up to four months after the procedure.^71, 72^ The prolonged presence of inflammatory markers suggests the possibility of increased HIV-1 in cervicovaginal secretions throughout that period. Women are typically counseled to remain abstinent for one month after treatment with LEEP to reduce the potential risk of infection or bleeding. A study from South Africa found that at least 50% of participants reported resumption of sexual intercourse within two weeks of undergoing LEEP or cryotherapy for treatment of cervical dysplasia.^36^ Increased HIV-1 secretions in the genital compartment after treatment for cervical dysplasia, combined with high levels of early post-treatment intercourse could place a significant number of partners at higher risk of HIV acquisition. To our knowledge, there has been only one study evaluating HIV-1 genital shedding after treatment for cervical dysplasia. That study by Wright, et al, showed an average *increase* of up to 10,000 copies of HIV-1 RNA in the cervicovaginal secretions post-treatment, with less than 1% attributable to the presence of blood in the genital tract.^73^ There were only fourteen women in the study, three of whom had cryotherapy and eight who had undergone LEEP. Gains in efficacy against cervical dysplasia may be offset by increased risk for infectivity if a method is associated with significantly greater duration and magnitude of HIV genital shedding.

The study described below will provide important information that can be used in the design and prioritization of an effective, resource-appropriate screening program. We seek to clearly define the test characteristics of two novel, but very different screening approaches that can be employed in resource-limited settings. We will then determine the magnitude of difference in HIV genital shedding between women with and without CIN2/3, and then explore the effect of diagnosis of as well as treatment for CIN2/3 on that shedding. The information gained from the research we carry out will provide a critical foundation in the effort to safely and effectively diagnose and treat cervical dysplasia among HIV-infected women in resource-limited settings, working toward prevention of cervical cancer in this population.

**JUSTIFICATION**

HIV and cervical cancer are diseases that disproportionately affect women in resource-limited settings. The challenge of reducing cervical cancer in developing countries is too complex to be overcome simply by introducing current detection methods from resource-rich countries into resource-limited areas. The infrastructure—facilities, equipment, and personnel—and training required for conducting a proper examination, collecting a high-quality sample, transporting the sample to a well-trained lab and adequately performing the tests present significant barriers that hamper effective implementation of known screening technologies in low-resource settings. Novel strategies that have received attention recently include VIA and biomarker testing, for the benefits outlined above. VIA has been widely studied and even implemented in resource-limited settings, but has not yet been validated among HIV-infected women. Testing for p16^INK4a^ has never been carried out among HIV-infected women in resource-limited settings, where it could have the most impact. We will define the test characteristics and identify a cut-off value for “positive” in this population, using a biochemical assay which has the potential to be developed into a point of care test. In addition, we will compare p16^INK4a^ test performance with HR-HPV which is the most commonly known biomarker available. The high prevalence of HR-HPV among HIV-infected women in Kenya makes us believe that this test would have low specificity for CIN2/3.^79^ It is important to compare the two currently available biochemical tests side-by-side, to inform clinical decisions and development of screening strategies in this population.

HIV-infected women are at increased risk for HR-HPV-related cervical dysplasia and cervical cancer, yet the impact of HR-HPV and cervical dysplasia on HIV-infectivity is relatively unknown. The evidence for increased HIV-1 genital shedding in the presence of other sexually transmitted infections or inflammation, along with the preliminary data on HR-HPV and CIN in HIV-infected women suggest that these may also increase HIV-1 genital shedding. This study improves on the preliminary work mentioned above in several ways. We will exclude women with concomitant cervical or vaginal infections, which may have independent effects that cannot be controlled for in the analysis. Our study population will include women on HAART, reflecting the increased numbers of women now on treatment. Finally, we will use CIN rather than HR-HPV as a predictor. CIN is well-accepted to be the result of persistent HR-HPV infection and clinical decision-making regarding treatment is based on the diagnosis of CIN, not HR-HPV.^80, 81^ The high HIV and HR-HPV co-infection rate in Kenya and the high proportion of HIV-serodiscordant partnerships in Kisumu make this study both feasible and important for this population. The FACES/UCSF relationship and the existing well-accepted cervical cancer screening program both combine to facilitate study-related activities. We also seek to provide researchers and clinicians with information about the potential increase in HIV-infectivity related to diagnosis of and treatment for cervical dysplasia, which could have an immediate impact on recommendations for length of post-procedure abstinence among women whose partner’s HIV status is negative or unknown. It will also be useful in the design of future studies to compare the efficacy and safety of different diagnostic and treatment modalities for cervical dysplasia among HIV-infected women. Finally, data about magnitude and duration of increased HIV shedding can be added to decision analysis models to estimate the impact, cost-benefit and cost-effectiveness of various treatment methods.

**HYPOTHESES (NULL)**

1. VIA, VILI and p16^INK4a^ will perform equally well as screening tests for cervical cancer in HIV-infected women.
2. Women with CIN2/3 will have the same frequency and quantity of HIV-1 RNA detected in their genital secretions as women without CIN2/3.
3. Women treated with LEEP will have the same quantity of cervicovaginal HIV-1 RNA detected pre- and post-treatment and up to 14 weeks after the procedure.

**OBJECTIVES:**

General:

We seek to establish the link between the presence, diagnosis and treatment of cervical dysplasia and HIV genital shedding. We also seek to establish the sensitivity and specificity of three resource-appropriate screening methods, VIA, VILI and p16^INK4a^ biomarker testing, for cervical dysplasia.

Specific (Aims):

**Aim 1:** **To calculate the sensitivity, specificity, and the positive and negative predictive values of visual inspection of the cervix after application of 3-5% acetic acid (VIA) and visual inspection with Lugol’s Iodine (VILI) for the diagnosis of cervical intraepithelial neoplasia grade 2 and grade 3 (CIN2/3) in HIV-infected women.** HIV-infected women enrolled in care at the FACES clinics in western Kenya will undergo VIA or VILI and colposcopy with directed biopsy as indicated. The colposcopy will be used as the reference standard, allowing for calculation of sensitivity, specificity, positive and negative predictive value of VIA and VILI.

Hypothesis 1: VIA and VILI will have test characteristics validating their use as a screening test for cervical dysplasia among HIV-infected women in resource-limited settings.

**Aim 2: To define the quantitative value of a biochemical assay for p16^INK4a^ that predicts cervical neoplasia with the greatest sensitivity and specificity among HIV-infected women using a receiving operating characteristic (ROC) curve.**

**Aim 2a: To compare the sensitivity and specificity of the p16^INK4a^ biochemical assay with that of HR-HPV testing.** HIV-infected women enrolled in care at the FACES clinics in western Kenya will undergo specimen testing for p16^INK4a^ and HR-HPV, followed by colposcopy with directed biopsy as indicated.

**Aim 2b: To calculate the sensitivity and specificity of the p16^INK4a^ biochemical assay for the detection of CIN2/3 using colposcopy as the gold standard.** HIV-infected women will have p16^INK4a^ specimens collected prior to colposcopy. Diagnosis for positive p16 will be based on the ROC curve cut-off determined in the pilot study, with colposcopy as the reference standard as above.

**Aim 2c: To investigate whether presence of increased baseline and follow-up levels of p16^INK4a^ in women with normal colposcopy or CIN1 is associated with increased risk of detecting CIN2/3 during one-year follow-up.** HIV-infected women enrolled in care at the FACES clinics in western Kenya who have had baseline p16^INK4a^ measurement, with normal colposcopy or CIN1 will be followed-up after one-year for repeat testing for p16^INK4a^, HR-HPV and colposcopy to compare rate of CIN2/3 between the two groups.

Hypothesis 2: The ROC curves for the biochemical assay for p16^INK4a^ will define a cut-off level for a positive result that will provide a sensitivity and specificity for CIN2/3 greater than those for HR-HPV testing in this population.

Hypothesis 2c: The risk of detecting CIN2/3 at one-year in women with normal colposcopy or CIN1 is increased in women with increased levels of p16^INK4a^ in cervical specimens at baseline and/or follow-up compared to women with normal or undetectable levels of p16^INK4a^.

**Aim 3: To measure and compare the quantity of HIV-1 RNA in the cervicovaginal secretions of women with cervical dysplasia to those with normal cervical exams.** We will measure and compare the presence and levels of cervicovaginal HIV-1 in women with biopsy-confirmed high-grade cervical dysplasia to those of women with no evidence of cervical disease. Cervical dysplasia will be defined as cervical intraepithelial neoplasia 2 or 3, or CIN2/3.

**Aim 3a: To determine the amount and duration of increased HIV-1 RNA shedding in the genital tract following cervical biopsy for suspected dysplasia.** We will measure the presence and levels of cervicovaginal HIV-1 in women prior to undergoing cervical biopsy and compare values to specimens taken on days 0, 2 and 7 following biopsy.

Hypothesis 3: Women with biopsy-confirmed CIN2/3 have higher odds of detectable genital HIV-1 viral load. Women undergoing biopsy will have an increase in genital HIV-1 RNA shedding that will return to baseline within 7 days post-procedure.

**Aim 4: To measure and compare the amount of HIV-1 RNA found in the cervicovaginal secretions in women before and after undergoing treatment for CIN2/3 with LEEP.**  Collection of specimens for detection and quantification of cervicovaginal HIV-1 viral load will be done immediately prior to the LEEP, and on post-procedure weeks 1, 2, 4, 6, 10 and 14 to determine the level and duration of increased genital shedding associated with the procedure.

Hypothesis 4: There will be a significant increase in HIV-1 genital shedding post-treatment which will return to baseline before 14 weeks post-treatment.

**DESIGN AND METHODOLOGY**

Study Site: This study will take place in FACES-supported clinics in the Nyanza Province of Western Kenya.

Study Population: The main target population for the proposed research is HIV-infected women enrolled in care at FACES-supported clinics in Nyanza Province.

Subject recruitment and enrollment: Women receiving care at FACES clinics will be considered for recruitment into the study. FACES patients are introduced to cervical cancer screening as part of an HIV education session when they enroll in care. Information about cervical cancer and the opportunity for screening is reinforced as part of a general daily health talk given while patients are waiting to be seen for their routine HIV care, and then reinforced through individualized counseling during their clinical visit. Women willing to undergo cervical cancer screening will be screened consecutively for their study eligibility and asked about their willingness to participate in the study. Inclusion and exclusion criteria are listed below:

| **Inclusion Criteria** | **Rationale** |
| --- | --- |
| Enrolled in HIV care at FACES | Allows for follow-up and shared utilities of space and personnel with FACES program |
| ≥ 23 yrs old | Based on WHO recommendations for established cervical cancer screening programs in resource-limited countries |
| Able to verbalize understanding of study procedures and provide informed consent | Ensure participant understanding and avoid potential coercion |
| *Additional Criteria for Specific Aim 2c:* |  |
| Participated in p16^INK4a^ ELISA pilot study and had a colposcopic finding of CIN1 or normal-appearing cervix ? | These women already have a baseline p16^INK4a^ biomarker level measured, hence qualify for follow-up study  Women with CIN2/3 would have undergone surgical treatment for their lesion, thus reducing their risk of disease at 1 year |
| *Additional Criteria for Specific Aim 3:* |  |
| On ARVs for at least 6 months with adherence >90% | Reflects majority of FACES clients and reduces confounding effect of ARVs on HIV-1 genital shedding^82^ |
| *Additional Criteria for Specific Aim 3a:* |  |
| Will undergo biopsy for positive colposcopic findings at one of the FACES study clinics in Kisumu |  |
| Willing to remain abstinent from sexual activities for one week post-biopsy | Reduces potential semen contribution to genital HIV-1 viral load measurements. |
| *Additional Criteria for Specific Aim 4:* |  |
| Will undergo LEEP for CIN2/3 at one of the FACES study clinics in Kisumu | Allows baseline specimen collection and ensures the quality and standard of the LEEP |
| Willing to remain abstinent from sexual activities for four weeks post-LEEP and for three days prior to each follow-up study visit | Reduces risk of post-LEEP bleeding and infection. Reduces potential semen contribution to genital HIV-1 viral load measurements. |
| Exclusion Criteria | Rationale |
| Pregnant | May increase false positive rate of VIA^75^ |
| Clinical evidence of cervical or pelvic infection:   - Muco-purulent discharge - Cervical friability - Cervical motion tenderness found on pelvic exam in symptomatic women | May increase false positive rate of VIA^75^ |
| Cervical cancer screening at FACES within past 12 months | May influence clinician interpretation of results |
| Prior or current history of cervical surgery, dysplasia or cervical cancer | May influence test characteristics of colposcopy and VIA |
| Unsatisfactory VIA | Woman would require alternative screening method (cytology, colposcopy or endocervical sampling) per WHO protocol^74^ |
| *Additional Criteria for Specific Aims 3 & 4:* |  |
| Laboratory evidence of cervical infection:   - NAAT for Gonorrhea or Chlamydia - Wet mount showing bacterial vaginosis, candida or trichomonas | Concomitant sexually transmitted infections can increase the risk of HIV-1 genital shedding |

We will sequentially recruit and enroll eligible women for all specific aims. There will be three separate consent forms (Appendix A, B and L). Screening and informed consent for participation in the studies involving VIA or VILI, colposcopy and specimen collection (S.A. 1, 2 and 3) will take place in a private gynecologic exam room prior to pelvic exam. Because satisfactory visual exam or clinical evidence of cervical or pelvic infection will only become evident after exam, participant numbers will be assigned after VIA and colposcopy. Women who are unsure about enrollment and would like time to consider will be given an information sheet (Appendix C) and may return for enrollment at a later date. VIA in these women will be deferred until their next visit. Eligibility and informed consent for the post-LEEP genital shedding sub-study will take place at a clinical visit, prior to their LEEP procedure. Women will be given an information sheet specific to this part of the study, in addition to the normal clinical materials in use that explain the LEEP procedure (Appendix D).

Statistical Considerations and Sample Size Calculation

**Specific Aim 1:** To calculate a sensitivity of 75% with a confidence interval of 5% in both directions and a two-sided alpha of .05, we will need to complete sequential VIA and colposcopy in ***1800* women**.^83^

To determine a specificity of 80%, with a confidence interval of 5% in both directions and a two-sided alpha of .05, we calculate that we will need to examine 400 women. Because the scientific outcome of specificity is most important, we will use the estimate of 1,800 women (900 VIA and 900 VILI to account for laboratory and sampling error) to power our study.

**Specific Aim 2:** We based our sample size calculations for this aim on the following calculations: a sample of 42 women with positive results and 60 with negative results will achieve 90% power to detect a difference of 0.1 between the histology result and the AUC for the p16^INK4a^ assay using a two-sided z-test at a significance level of 0.05.^84^ Histology is the gold standard for diagnosis of CIN, which would give it an AUC of 1.0; a 0.1 difference mean an AUC for p16^INK4a^ of 0.90, which would make it an excellent diagnostic tool. Our power to detect even greater difference with this sample size would be even greater. We will enroll ***60* women with negative colposcopies, and *140* women with positive colposcopic exams**. The positive predictive value of a positive colposcopy at FACES is approximately 57% for CIN2/3 and 89% of any diagnosis of CIN.^85^ A sample of 140 women with positive colposcopic exams will ensure that we have an adequate number of women with the positive result of interest (CIN2/3) to achieve our specific aim, and potentially enough women with lower levels of CIN to obtain preliminary information regarding test characteristic among this group. In order to calculate the specificity and sensitivity (SA 2b), we will use the sample sizes calculated above for specific aim 1.

Specific Aim 2c: To investigate whether increased levels of p16^INK4a^ in women with normal colposcopy or CIN1 puts them at higher risk for detecting CIN2/3 after one year, we use an effect size from literature to calculate our sample size. This question has not been previously studied in HIV+ women in resource-limited settings. A previous study of HIV-negative women in North America found that women with CIN1 with diffuse staining for p16^INK4a^ had between 26.9 – 33.6% increased risk of CIN2/3 detection during follow-up compared to those with weak or no staining for p16^INK4a^.^17^ Using this effect size, with a two sided alpha of .05 and power of 80 percent, we will need between **39 - 55 subjects with CIN1 diagnosis** in each group (with and without elevated p16^INK4a^) to show a similar or greater effect size (total 78-110 participants). Wang et al (2004) looked at both women with normal colposcopy and CIN1, and found that those with positive p16^INK4a^ staining at baseline had a 23.8% higher rate of progression to CIN2/3 during follow-up compared to those with negative staining.^16^ Using this effect size to estimate sample size, we need to study 82 subjects with normal colposcopy each in the biomarker positive and biomarker negative groups (total 164) to establish a similar effect size with 80% power. From the p16^INK4a^ pilot study, we have a total of 55 women found to have CIN1 and 190 women who had normal colposcopy, with about half of each group having increased levels of the biomarker. Based on this, we will be **a bit shy of the 39-55 participants** needed in the CIN1 group to show a similar effect size as the study quoted. However, we believe we can still be able to show a smaller yet clinically important effect size. For participants with normal colposcopy at baseline, we have sufficient number of women from the pilot study with this finding, such that with aggressive follow-up we should be able to show a similar or greater effect size as that seen in literature. Because this is an exploratory study, even if we are unable to achieve power for some of the sample sizes we are using, the results will be important for suggesting further research.

**Specific Aim 3:** Our sample size calculation is based on the frequency of cell-free HIV-1 RNA detected among women with CIN or HR-HPV in the two studies mentioned above. In the earlier study of HAART naïve women by Spinillo, et al, 81% of women with HR-HPV had measurable HIV-1 RNA in their cervicovaginal secretions compared to 55% of women without HR-HPV (OR 8.63).^86^ In a second study, by the same authors, 40% of women with CIN had measurable HIV-1 RNA in their cervicovaginal secretions, compared to 17% of women without CIN (OR 1.86).^68^ Extrapolating from the results of these studies, we anticipate a difference of HIV-1 detection frequency between cases and controls of approximately 25%; we would consider a difference of this magnitude to be clinically significant. To obtain a power of 80% with a two-sided alpha of 0.05 to detect this difference, we would need to enroll 46 women per study arm.^87^ In order to ensure that we have sufficient power, we will **enroll *50* women in both arms**. Aim 3a is an exploratory pilot study from which we hope to obtain descriptive data to inform future studies. Therefore we did not calculate a sample size. We will evaluate 30 women for this study, using guidance from the sample size calculation listed below.

**Specific Aim 4:** Sample size calculations for Aim 4 focus on estimation of the mean change in viral load between baseline and one week of follow-up. A sample size of 30 will provide sufficient power to address hypotheses of interest, and will also allow investigation of factors related to observed changes in viral load. We assume a standard deviation of 1.7 log copies for both the baseline and follow-up measure, that the correlation between the baseline and follow-up measure will be 0.7, and that change will be evaluated using a t-test for paired measurements. This calculation is based on variability estimates from Coombs et al., who examined genital tract shedding in 55 women using Sno-Strips, cytobrush and cervicovaginal lavage measures. They found Sno-Stripscalc to be the least variable measure, with a within-individual standard deviation of 0.55 log copies of viral RNA and a between-individual standard deviation of 1.66 log copies. Although preliminary data are not available to inform more detailed calculations, we anticipate similar levels of power to address hypotheses concerning post-treatment changes. With these assumptions, ***30* participants** should provide at least 80% power to detect within-individual changes (measured as the difference between pre and post-treatment levels) as small as 1 log as significant at the 5% level. Power to detect change as small as 1.5 logs will exceed 95%. These results indicate that we have sufficient power to detect clinically important changes, and will also be able to investigate patterns of change over the entire course of follow-up.

Study Design

We propose a ***cross-sectional*** study to evaluate this specific aim. Each participant will undergo a VIA or VILI with specimen collection for p16^INK4a^ and HR-HPV and colposcopy by a different clinician during a single clinic visit. The study clinician performing the colposcopy will be blinded to the results of the VIA or VILI. For women in the VILI arm, the colposcopy will be performed first in order to eliminate any interference with colposcopy interpretation from the iodine staining. Biopsy decisions will be made at the time of colposcopy, but deferred until after the VILI. Any woman with cervical lesions suspicious for dysplasia seen at the time of colposcopy will undergo biopsy. Colposcopy will be used as the reference standard for presence or absence of CIN2/3, with biopsy results being the final diagnosis when available. Women with a normal colposcopy without biopsy will be considered to have no disease.Specific Aims 1, 2, 2a and 2b)

We propose a **prospective cohort** study using a subset of women identified from the cross-sectional study in Specific Aims 1 and 2a to evaluate specific aim 2c during a one-year follow-up visit. We will recruit women for this follow-up study who participated in the pilot p16^INK4a^ ELISA study (Aim 2a) and had normal colposcopy or CIN1. In a single visit, each participant will undergo a VIA with specimen collection for p16^INK4a^ and HR-HPV followed by colposcopy and biopsy if needed.

We propose a ***case-control*** study to measure and compare the presence and quantity of HIV-1 RNA in the cervicovaginal specimens of women with biopsy-confirmed CIN2/3 (cases) and age-matched women without evidence of cervical disease as measured by negative VIA (controls). We will compute the odds ratio for HIV-1 detection and compare log transformed mean viral loads between cases and controls. (Specific Aim 3)

A ***prospective case crossover*** study design will be employed to evaluate the magnitude and duration of increased HIV-1 RNA genital shedding after biopsy and LEEP. Participants undergoing biopsy and those with biopsy-confirmed CIN2/3 who undergo treatment with LEEP at FACES will be recruited. For aim 3a, specimen collection specimen collection for cervicovaginal HIV-1 RNA will take place prior to and immediately following cervical biopsy, and again on days 2 and 7 post-procedure. For S.A. 4, collection will take place prior to the LEEP and at 1, 2, 4, 6, 10 and 14 weeks post-procedure. Proportion of women with detectable HIV-1 genital shedding at each time point will be calculated and compared to baseline. Difference in absolute log values of HIV-1 RNA from baseline will be compared by individual and as a mean for each time point. (Specific Aim 3a and 4)

Study Procedures

**Pelvic Exam and Specimen Collection:** Study examinations will take place in a private room equipped with a gynecologic exam bed in the FACES clinic. Participants will be positioned in the dorsal lithotomy position. The external genitalia will be examined to look for any evidence of infection or genital ulcer disease. After introduction of a sterile speculum, the clinician will use a swab to wipe away cervical mucus or blood and assess for evidence of cervicitis.

- **Cervical specimens for p16^INK4a^ and HR-HPV:** A single Dacron swab will be placed into the cervical os, rotated twice and then swabbed over the ectocervix. The swab will then immediately be placed into a cryovial with normal saline. At the end of each day, specimens will be flash-heated to denature any enzymes that may work to break down p16^INK4a^.
- **Cervical specimens for STI testing with NAAT:** Following p16^INK4a^ and HR-HPV testing, a second Dacron swab will be placed in the cervical os and gently rotated for 10-30 seconds, removed, placed in the transport media. Specimens will be stored at room temperature at the FACES lab until analysis is performed.
- **Cervical specimens for HIV-1 RNA detection and quantification:** A study clinician will place 3 Sno-Strip™ wicks (Chauvin Pharmaceuticals Ltd., Essex, England) in the posterior fornix of the vagina and allowing the vaginal fluid to absorb to each strip. The vaginal fornix was chosen instead of the endocervix because vaginal Sno-Strips provide a more reproducible detection and quantification of HIV-1 RNA than endocervical Sno-Strips, especially in the presence of endocervical mucus and debris.^88^ We also want to avoid disruption of the post-procedure healing cervix. The Sno-Strips will be placed in NASBA buffer and processed by the on-site laboratory within 2 hours of collection. After this time they will be placed in the –80º freezer until transport to the ARI-UCSF Laboratory of Clinical Virology for viral load testing.
- **Vaginal specimens for yeast, bacterial vaginosis and trichomonas:** After cervical specimen collection is complete, the study clinician will use a cotton swab to collect any fluid or discharge from around the posterior fornix and vaginal walls and place it in a test tube with 2cc of normal saline. The clinician will read the specimen immediately after the study examination and discard it after recording the results.

**Visual Inspection with Acetic Acid:** After identification of the cervical anatomy, including the squamo-columnar junction, the clinician will apply 5% acetic acid. After one to two minutes, the clinician will examine the cervix again to interpret the results of the VIA.

VIA will be defined as negative if the following findings are present:

- circumferential identification of the squamo-columnar junction (SCJ)
- no aceto-white lesions or faint, ill-defined, bluish white lesions at the SCJ or aceto-white lesions far away from the SCJ

VIA will be defined as positive if the following findings are present:

- well-defined, opaque aceto-white lesions touching the SCJ or surrounding the external os
- large circumferential aceto-white lesions covering the external os,
- pre-existing condyloma or leukoplakia which turns intensely white after application of acetic acid

**Visual Inspection with Lugol’s Iodine:** After identification of the cervical anatomy, including the squamo-columnar junction, the clinician will apply Lugol’s Iodine. After staining is noted in the transformation zone, the clinician will examine the cervix again to interpret the results of the VILI.

VILI will be defined as negative if the following findings are present:

- circumferential identification of the squamo-columnar junction (SCJ)
- complete iodine uptake around the SCJ and into the transformation zone

VILI will be defined as positive if the following findings are present:

- well-defined, iodine yellow areas within or immediately surrounding the SCJ
- patchy areas of missing iodine uptake in the transformation zone

In order to maintain strict blinding of study clinicians, the results of the VIA or VILI will not be shared with the participant until after the colposcopy has been performed. The study clinician performing the VIA or VILI will leave the room with the data collection form containing the test results before the second clinician enters the room to perform the colposcopy.

**Colposcopy and biopsy:** Colposcopy will take place by a second, blinded study clinician immediately after VIA or VILI. Colposcopic exam will be done in accordance with the International Federation for Cervical Pathology and Colposcopy (IFCPC) guidelines,^89^ and will include sequential evaluation using green filter, acetic acid and Lugol’s Iodine. Findings on colposcopy that are suggestive of a CIN2/3 or worse lesion will prompt a colposcopically-guided biopsy. A normal colposcopy will be considered a final result indicative of no disease. (What about CIN1?) *the hgcin should have gotten a biopsy. The clinician performing the colposcopy will be assisted by the study nurse, who will also be blinded to the results of the VIA. They will have access to the participant’s clinical record, but not to the VIA results. Colposcopy findings will be recorded on a separate data collection form. After the colposcopy findings have been recorded the results of both tests will be discussed with the participant, including implications for treatment and future screening.

Study visits (Appendix E)

Specific Aims 1, 2a, 2b and 3:

**Enrollment visit**: Informed consent, VIA/VILI, colposcopy and genital specimen collection for HIV-1 viral load, HR-HPV, p16^INK4a^, and STI testing will take place during a single visit. If indicated by abnormal findings at colposcopy, participants will undergo directed biopsy at the time of colposcopy for diagnosis of cervical dysplasia. No biopsies will be performed solely for study purposes. Follow-up appointments will be made for two to four weeks after enrollment visit among participants undergoing biopsy. This visit will comprise the total study participation for subjects in the cross-sectional study who have negative colposcopy. They will be scheduled for their regular clinical follow-up as indicated by their health status.

For specific aim 3, matching of controls to cases will take place after cases have been identified. The inclusion criteria for the case-control study depend on test results that are not readily available (negative STI testing and biopsy results positive for CIN2/3), therefore cases will not be identified until their follow-up visit. In order to minimize confounding secondary to biopsy collection, HIV-1 viral load specimens for cases and controls will be taken during the single study visit for specific aims 1, 2 and 3. Samples will be kept in the on-site –80º freezer until the time that cases are identified, at which time samples from women ineligible for enrollment will be discarded.

**Follow-up Visits at day 2 and 7:** Women who underwent cervical biopsy and are eligible for S.A. 3a will return on day 2 and 7 following biopsy, during which time they will undergo a sterile speculum exam with specimen collection for HIV-1 RNA.

**Results Visit (for clinical care, not required for study participation):** Women undergoing cervical biopsy will be scheduled for a follow-up visit in two to four weeks. They will receive their biopsy results from the study clinician, and may be offered enrollment into the post-LEEP HIV-1 shedding study if eligible.

Specific Aim 2c:

**Single Follow-up visit**: Informed consent, VIA, colposcopy, and cervical specimen collection for HR-HPV, and p16^INK4a^ will take place during a single follow-up visit for women who previously underwent p16^INK4a^ testing and meet criteria for these specific aims. If indicated by abnormal findings at colposcopy, participants will undergo directed biopsy at the time of colposcopy for diagnosis of cervical dysplasia. No biopsies will be performed solely for study purposes.

Specific Aim 4 (7 study visits):

**Enrollment:** Women returning for their biopsy results who have CIN2/3 will be offered treatment with LEEP at this visit, as is the current clinic protocol at FACES. Recruitment and enrollment into the post-LEEP HIV-1 shedding study will take place at this visit. Women who meet eligibility criteria will be offered enrollment. Informed consent for this study will be obtained during this visit, and participants will be assigned a study ID number. A follow-up visit schedule will be made.

**Follow-up Visits at 1, 2, 4, 6, 8, 10 and 14 weeks:** Participants will undergo a brief interview followed by a sterile speculum exam with specimen collection for genital HIV-1 RNA.

Study measurements

Individual participant data, including demographic and clinical information will be collected from the paper medical record using study-specific data collection forms. Individual and de-identified aggregate data will be abstracted from the electronic medical record system (OpenMRS) in place at FACES. Participants will all be enrolled in FACES for care of their HIV disease; as part of this care, they have regular laboratory monitoring of CD4+ counts, hemoglobin levels and basic serum chemistries. Laboratory data for the study, apart from histology and HIV-1 RNA specimens, will be obtained from their FACES charts, and will not be collected independently for the study.

**Predictor Variables**

Predictor demographic variables will include participant age, gravidity, parity, history of cervical cancer screening, marital status and number of current and previous sexual partners. Clinical predictors will include WHO status of HIV disease, CD4+ count, opportunistic infections, previous sexually transmitted infections and current use of anti-retroviral therapy.

For Specific Aim 2c, predictor variable will be p16^INK4a^ ELISA levels found during initial screening. The other variables above will be collected and treated as potential confounders.

**Outcome variables**

**Specific Aim 1:**

- VIA or VILI result (positive or negative)
- Colposcopy result (absence of CIN2/3) or result of colposcopically-directed biopsy if indicated for suspicion of CIN2/3, as is commonly used reference standard for cervical cancer screening studies.^90, 91^

**Specific Aim 2:**

- p16^INK4a^
- HR-HPV (types: 16, 18, 31, 33, 35, 39, 45, 51, 52, 56, 58, 59, 68)
- Colposcopy result (absence of CIN2/3) or result of colposcopically-directed biopsy if indicated for suspicion of CIN2/3

Specific Aim 2c:

- Colposcopy result (absence of CIN2/3) or result of colposcopically-directed biopsy if indicated for suspicion of CIN2/3

**Specific Aim 3:**

- Presence and quantity of HIV-1 RNA viral load in cervicovaginal secretions at the time of VIA/colposcopy. HIV-1 quantity will be expressed as log copies per microliter (µL) of vaginal fluid.

**Specific Aim 4:**

- Presence and quantity of HIV-1 RNA viral load in cervicovaginal secretions at each of the study time points.

**ETHICAL CONSIDERATIONS**

Human Subjects Involvement and Characteristics

The study described in this proposal will involve a total of around 1,800 HIV-infected women receiving care at the FACES clinics in Kisumu, Kenya. Women will be included in the study for the cross-sectional validation study if they are: 1) 25 years or older 2) not pregnant 3) accept cervical cancer screening 4) have a satisfactory Visual Inspection with Acetic Acid (VIA). Participants will be recruited for the prospective follow-up study (Specific Aim 2c) if they meet the above criteria, and were found to have normal colposcopy or CIN1 upon initial screening with p16^INK4a^ biomarker measurement. Participants will be recruited for the prospective post-LEEP HIV-1 shedding study if they meet the above criteria, have undergone LEEP for treatment of CIN2/3 and agree to remain abstinent for three days prior to each of the follow-up visits. To participate in any of these study activities, women must be willing and able to participate, be able to sign or mark a consent form, and speak one of the languages into which the consent form and data collection instruments have been translated, including English, D’Luo and Kiswahili.

Sources of Materials

Data for this study will be collected from several sources. Clinical and demographic data will be obtained through chart reviews and physical examination. We will also obtain clinical information from the colposcopic examinations. Information from the colposcopy and if performed, biopsy, will be entered into the participants’ medical records as it will be used to direct clinical care. We will also obtain cervical specimens for p16^INK4a^ and HR-HPV testing and vaginal wick specimens for women in the post-LEEP HIV-1 shedding study. These will be obtained solely for research purposes, and will therefore not be entered into the participant’s medical record. All data collection will take place in a private study room.

Potential Risks

We anticipate that the potential risks to participants in the study will be minimal. Study subjects may feel discomfort or embarrassment during the pelvic exam. Participants may be at risk for psychological or emotional discomfort at different stages of the study if they have a diagnosis of CIN2/3 and learn about potential risk of cervical cancer. Participants may risk some discomfort and bleeding after the colposcopy and biopsy, if performed. We anticipate that this will be minimal, as exams will be performed in a very gentle manner by trained clinicians. We do not anticipate physical discomfort from the collection of cervical specimens, however participants in the prospective HIV-1 shedding study may risk some post-LEEP bleeding after the speculum exam at the follow-up study visits. We anticipate that this will be minimal, as speculum exams will be performed in a very gentle manner by trained clinicians and we do not anticipate physical discomfort from the collection of Sno-Strip specimens during the follow-up visits.

Adequacy of Protection Against Risks

To reduce the likelihood of participants experiencing psychological discomfort, we will make every effort to create a secure and trustworthy environment. Study staff that counsel participants and carry out examinations will undergo training on culturally sensitive ways to reduce discomfort and embarrassment during the pelvic exam. To assist participants who may experience psychological discomfort when learning of a diagnosis of cervical dysplasia, we will ensure access to experienced counselors who work at FACES. Participants will be screened for signs of depression or difficulty coping with the added diagnoses at study visits.

All study staff will be thoroughly trained in the study protocol, study procedures, good clinical practices and protection of human subjects prior to starting the study. All exams will be performed gently and in a way that most minimizes discomfort, bleeding or anxiety in the participants.

Recruitment and Informed Consent

Women will be recruited in the cervical cancer screening rooms in the FACES clinics at Lumumba and Kisumu District Hospital. Upon enrollment into care at FACES, all patients take part in an HIV education course, which includes an introduction to cervical cancer risk and opportunities for screening. Health education talks occur daily in the clinic waiting area; among the covered topics are cervical cancer screening. Women who are eligible for cervical cancer screening are counseled in private by a triage nurse prior to their clinical visit. After their clinical visit, women who agree to screening are seen in a private gynecologic examination room. A trained study nurse will take women who meet the eligibility criteria through an informed consent process. As part of the informed consent process, potential participants will be informed of the purpose and methods of the study, procedures to protect the confidentiality of their clinical and study related information, their right to withdraw from the study at any time, the fact that their participation or non-participation will not affect the medical care that they receive at FACES, and persons to contact if they have questions about the study after completion of the study visit. The women will also be asked permission to obtain information from their medical records. Study subjects will be provided with a copy of the entire consent as well as an information sheet in simple language to take home. Participants may provide verbal consent and may place their mark on the consent form, if they are unable to provide a signature. No study procedures or data collection will take place prior to obtaining informed consent.

For Specific Aim 2c, we will prospectively recruit women who participated in p16^INK4a^ ELISA screening in the last one-year and who where found to have a normal colposcopy or CIN1. For the women found to have CIN1 at initial screening, per current protocol they would be due for a repeat colposcopy after one year. These women will be recruited into this follow-up study when they present for their clinically indicated colposcopy visit. For the remainder of eligible women who were found to have normal colposcopy at initial screening, they will be recruited for this follow-up study separately as they present for their routine medical visits. For these women, repeat screening would not be indicated at the one-year follow-up and will be done only for study purposes. We recognize that there will be a potential risk of lost confidentiality because women in this group will be identified based on their previous study participation. We will take measures to ensure the patients’ privacy is protected, such as only using patient ID numbers to flag files of eligible study participants, rather than using patient’s names.

Protection Against Risk

UCSF’s Institutional Review Board, the Committee on Human Research, and KEMRI’s Steering Committee, Scientific Steering Committee and Ethical Review Committee will review this protocol prior to study initiation. I will ensure that all procedures conform to US, Kenyan and international ethical standards regarding research involving human subjects. Guidance from Dr. Bukusi, the site-PI at FACES, will be critical in assuring that the protocol meets strict Kenyan standards for protection of research subjects. We will also seek assistance from the FACES Community Advisory Board (CAB) comprised of a diverse group of members of the Kisumu community, including FACES patients. The CAB will meet with members of the research team bimonthly to quarterly throughout the study to ensure that the study team is sensitive to potential sources of risk and discomfort to study subjects.

All participants will be assigned a unique alphanumeric code. One file linking the study identifiers with individual names will be kept by the Principal Investigator in a secured computer file. The file will be destroyed after data analysis is complete. All study forms will be maintained in a locked file cabinet in the FACES clinic.

Potential Benefits of the Proposed Research to the Subjects and Others

The research described in this plan will not provide any direct benefit to the participants, beyond a potential increase in knowledge about cervical cancer screening and prevention gained from spending more time with study staff and study clinicians. According to KEMRI regulations, participants will only be reimbursed for their transportation expenses related to participation in the study. Women enrolled in the cross-sectional/case-control portion of the study will not require an extra study visit, therefore they will be reimbursed with a meal voucher only. Women who provide consent and enroll in the longitudinal study will receive a transportation reimbursement of 300Ksh, in accordance with the level set by KEMRI.

Importance of the Knowledge to be Gained

The research plan described above will provide important information about an effective, resource-appropriate cervical cancer screening and prevention strategy that will help reduce the incidence and mortality from cervical cancer among HIV-infected women. This may have the potential to improve access for women who are at high-risk for the disease both biologically and because they live in settings where they have limited access to care. The information from the post-LEEP HIV-1 shedding study will help inform future research, specifically, a randomized trial to evaluate cervical cancer screening and treatment in resource-limited settings. Additionally, information about the amount and duration of post-LEEP HIV-1 shedding may change the understanding of post-LEEP infectivity and counseling for post-LEEP sexual activity, ultimately affected HIV transmission rates. Together these aims comprise the next step in the effort to accurately diagnose and safely treat cervical dysplasia among HIV-infected women in resource-limited settings.

Privacy and Security of Health Records

All paper and electronic medical records for participants and potential participants in this study will be secured and maintained in compliance with UCSF campus administrative policy 650-16.

**DATA QUALITY AND MANAGEMENT**

Staff Training: Study exams will be carried out by clinical officers and nurses after undergoing training in speculum exam, VIA, specimen collection, colposcopy and directed biopsy. The training will be based on the International Agency for Research on Cancer,^75^ PATH and WHO^74^ curricula and will consist of a series of didactic lectures, discussions, review of cervical photographs and hands-on experience. All study staff will undergo training in Good Clinical Practices, informed consent, participant confidentiality and study-specific procedures. After satisfactory completion of training, including an examination and proctored procedures, staff will receive certification. Periodic training and assessment sessions will be done throughout the study. Lab technicians will be oriented in the proper handling, storage and transport of laboratory specimens.

Quality Assurance: The main study measurements which will be subjected to quality assurance measures to eliminate the risk of random error and measurement bias: VIA and colposcopy reading, interpretation of histopathology specimens, p16^INK4a^ levels, HR-HPV reading and measurement of HIV-1 viral load. The quality of VIA and colposcopy is dependent on the training, supervision and periodic assessment of the clinical officers performing the examinations. To further reduce random error, all colposcopies will be interpreted using the guidelines of the IFCPC as has been done in prior studies.^89^ Histopathology specimens will be read by a trained cytopathologist at KEMRI. The first 20% of specimens will be double read by a pathologist at UCSF, who will be blinded to the results of the KEMRI pathologist. Discrepancies will be clarified by consultation and review of the specimens by both pathologists. If no consensus if reached, the results will be reviewed by a third, blinded UCSF pathologist.

The main outcome for this specific aim will be p16^INK4a^ values. Monoclonal antibody testing for p16^INK4a^ will be performed on all specimens using an ELISA-based biochemical assay currently available only in specified research laboratories. Samples for the p16^INK4a^ ELISA will be transferred into a collection vial with a prototypic sample lysis medium (MTM Laboratories, Heidelberg, Germany) containing nonionic detergents (<1%). Lysed samples will be stored frozen until the ELISA-based measurement of p16^INK4a^ protein concentration is performed. Next, 100-µL aliquots of each individual sample were subjected in duplicate to a prototypic colorimetric sandwich-ELISA protocol (MTM Laboratories), which is based on two different p16^INK4a^-specific monoclonal antibodies, an antibody that was coated to the solid phase of a microtiter plate (clone E6H4) and an antibody that was conjugated to horseradish peroxidase for the detection of captured p16^INK4a^ protein (clone D7D7). For quantification, calibration curves will be established by using reagent standards that contained known concentrations of p16^INK4a^ antigen. p16^INK4a^ protein concentrations in the clinical samples were calculated as arbitrary units (U/mL) based on the standard curve. Average values of double measurements were used, and the coefficient of variation was <15% at a cutoff value of 16 U/mL. In order to ensure the reliability of the biochemical assay measurements, quantitative levels of p16^INK4a^ will be compared with levels found in immunohistochemical (IHC) staining of the corresponding biopsy specimens. Correlation will be determined through the calculation of a Kappa coefficient, which we seek to be greater than 0.8. In cases of normal colposcopy (and hence no biopsy), p16^INK4a^ IHC values will be assigned as zero. The IHC staining will be done by pathologists at KEMRI, who have experience in this technique and will undergo further study-specific training coordinated by Dr. Magnus von-Knebel-Doeberitz at the German Cancer Research Center in Heidelberg.

HR-HPV DNA testing will be done at the MTM laboratories, under Dr. von-Knebel Doeberitz’s direction. Testing will be done using fluid from the same collection vial as the p16 testing, and will be done using the Hybrid Capture 2 (Qiagen, Inc.) nucleic acid amplification test for 13 high risk HPV types 16, 18, 31, 33, 35, 39, 45, 51, 52, 56, 58, 59, 68. Results of the HPV DNA assay will be expressed as a ration of relative light units (rlu ratio) to the average of three positive controls, with 1.00 as the cut-off for test positivity. Internal quality assurance parameters and positivity cut-off parameters are determined by the manufacturer’s instructions. Positive HC2 tests will then be typed using reverse dot blot hybridization with recombinant HPV type-specific plasmids (Luminex, Inc). If the confirmatory test is negative, the amplimers will be cloned and sequenced. Only Hybrid Capture 2-positive samples confirmed by reverse dot blot hybridization or sequencing will be classified as HPV-positive.

The ARI-UCSF Laboratory of Clinical Virology, which will perform the HIV-1 RNA testing on genital specimens, is licensed by the State of California for clinical virologic and immunologic testing (CLIA # 05D0969871). The laboratory adheres to a comprehensive Quality Assurance program, and undergoes regular monitor visits from Laboratory Field Services and PPD, a contractor for the NIH/DAIDS, to ensure adherence to Good Clinical Laboratory Practices (GCLP) while supporting federally funded clinical trials.

Permits will be obtained through KEMRI prior to shipping specimens to UCSF and the MTM laboratories. We will continue to search for and work with local partners with laboratory capacity and expertise to run specimens for genital HIV-1 RNA. When genital HIV-1 RNA testing has been validated in local laboratories, we will run our specimens through those labs. We anticipate that this will be more efficient, less expensive and help to build local capacity. The p16^INK4a^ biochemical assays are not available at this time in Kenya, one of the reasons we are carrying out the work here. We are confident that the experience and results from this project will lead to further p16^INK4a^ testing and development of a point-of-care assay, work to be carried our by laboratories and clinical researchers within Kenya in the future.

Data Storage: The study will be conducted within the existing clinic settings in the Nyanza Province. Data will be collected from the electronic medical record and on study-specific forms (Appendices F and G). Study data will be included as part of the patient’s medical file and maintained in locked file cabinets within the clinic. Data will be abstracted to computers by data entry clerks and managed by the assigned data manager at each site. Abstracted data will be labeled by unique study ID only. A file correlating study ID and medical file number will be kept in separate password-protected Excel computer file and accessible only to the core research team. Additionally, all study computers are password protected and kept in locked offices within the clinics.

Statistical Analysis:

Statistical analysis for all specific aims will be carried out using STATA and SPSS software.

**Specific Aim 1**: The goal of specific aim 1 is to calculate the sensitivity, specificity and predictive values of VIA and VILI for CIN2/3, with colposcopy as the gold standard. Colposcopy will be performed on every participant, regardless of screening result, to avoid verification bias.^92^ To calculate the sample size for this study, we assume that the prevalence of CIN2/3 among HIV-infected women at FACES will be 7%, as has been seen in the cervical cancer screening program at FACES.^78^ Sample size calculations will be based on prior estimates of VIA sensitivity, as these are more prevalent and therefore have more precision.

Sensitivity: Previous studies have shown that VIA has a sensitivity of 60-86%^39, 40, 91, 93-97^ for detection of CIN2/3 among healthy women, which is comparable to the sensitivity of cervical cytology and would be an acceptable test characteristic for this setting. In order to determine sensitivity, we will construct a 2 x 2 table, and divide the number of women with a true positive VIA result (i.e. those with disease diagnosed by colposcopy and VIA) by the number of true positive VIAs plus the number of false negative VIAs. Specificity: Specificity values for VIA range from 64 to 94% for the detection of CIN2/3.^39, 40, 91, 98, 99^ To determine specificity of VIA, we will divide the number of participants with true negative VIA (i.e. has no disease diagnosed colposcopically) by the number of true negative tests plus the number of false positive tests.

In addition to sensitivity and specificity, we will calculate the positive and negative predictive values of VIA. Studies have shown a PPV of VIA for CIN2/3 to range from 9-27% in HIV-negative women.^97, 100, 101^ To calculate the PPV in our study, we will divide the number of participants with a positive VIA by the number of participants found to have CIN2/3 on biopsy. VIA has been shown to have a negative predictive value (NPV) of 91-99.5% in HIV-negative women.^102^ To calculate the NPV, we will divide the number of participants with a negative VIA by the number of participants with no evidence of disease at colposcopy, either by visual impression or a negative biopsy result. Confidence limits for estimates of sensitivity, specificity, PPV and NPV will be exact, based on the binomial distribution.

Finally, we will compare the test characteristics between the two screening modalities using a McNemar’s test of proportions.

**Specific Aim 2**: To determine the cut-off value for the p16^INK4a^ biochemical assay that will provide the highest sensitivity and specificity for the detection of CIN2/3, we will construct a Receiver Operating Characteristic (ROC) curves using the results of the ELISA-based assay and the histology specimens. An ROC curve will allow us to plot the True Positive Rate (TPR) and the False Positive Rate (FPR) for various values of the p16^INK4a^ assay. As TPR is equivalent to the sensitivity of a test, and 1-FPR is the specificity, the graphical representation, as well as the calculated Area Under the Curve will allow us to determine the cut-off value for p16^INK4a^ to define a “positive test” with the highest sensitivity and specificity.

We will compare the absolute differences in sensitivity and specificity for detection of CIN2/3 of p16^INK4a^ and HR-HPV using the Mann-Whitney U test. The values of p16^INK4a^ will be determined from the ROC curves in Specific Aim 2. The sensitivity and specificity of the HR-HPV tests will be calculated as below:

- To calculate **sensitivity**, we will divide the number of women with a true positive test by the number of true positives plus the number of false negative HR-HPV testing.
- To calculate **specificity**, we will divide the number of participants with no disease diagnosed colposcopically (true negatives) by the number of true negative plus false positive HR-HPV tests.

Confidence limits for estimates of sensitivity and specificity will be exact, based on the binomial distribution.

**Specific Aims 2c:** To compare the rate of detecting CIN2/3 at one-year follow-up between women with normal or increased levels of p16^INK4a^ at baseline, we will use the Pearson’s chi-square test to investigate any significant differences in the proportions. Based on our sample size calculations, we aim to have 80 percent power with a two-sided alpha of 0.05. To compare the rates of developing CIN2/3 at one-year follow-up between women with persistently elevated p16^INK4a^ compared to those with only one elevated measurement, we will also use the Pearson’s chi-square test to look for statistically significant differences in proportions of CIN2/3 in the two groups.

**Specific Aim 3:** To compare the presence and magnitude of HIV-1 genital shedding between women with and without cervical dysplasia we will compute the odds ratio of HIV-1 detection in genital secretions with 95% confidence intervals. Multivariate analyses will be performed using logistic regression to control for demographic and clinical confounders. Potential confounders include WHO stage of HIV disease, CD4+ count, plasma HIV-1 viral load, opportunistic infections, previous STIs and current use of anti-retroviral therapy.

**Specific Aim 4:** The goal of specific aim 4 is to determine the magnitude and duration of increased HIV-1 genital shedding that occurs after treatment with LEEP. Observed time trends in log copies of genital HIV-1 RNA will initially be evaluated via exploratory analyses of longitudinal measures based on scatter plot smoothing methods. Results will allow us to assess possible nonlinearities in trends, as well as investigate differences in results between subgroups of interest. The subgroups we are looking at include participants on HAART and who have CD4+ counts below 200. Further analyses will focus on estimation of post-LEEP changes from baseline levels in log copies of HIV-1 RNA using linear mixed effects regression methods. The primary predictor will be time on study (measured in weeks post-treatment). Initial models will include separate coefficients for each week of observation post-treatment (2, 4, 6, 10 and 14). Hypothesis 2 will be addressed directly via tests constructed about these coefficients. We will also explore the effect on results of adjustment for selected covariates, including demographic characteristics and baseline descriptors of participant health status.

**TIME FRAME/DURATION**

|  | July-Sept 2010 | Oct-Dec  2010 | Jan-March  2011 | April-June  2011 | July-Sept  2011 | Oct-Dec  2011 | Jan-March  2012 | April-June 2012 |
| --- | --- | --- | --- | --- | --- | --- | --- | --- |
| Hire and train project coordinator | X |  |  |  |  |  |  |  |
| Develop SOPs, Study Manual, Database | X |  |  |  |  |  |  |  |
| Train clinic and lab staff in protocols | X | X |  |  |  |  |  |  |
| Participant Enrollment and Specimen Collection |  | X | X | X | X |  |  |  |
| Specimen Processing and Evaluation |  |  | X | X | X | X |  |  |
| Analyze data |  |  |  |  | X | X | X |  |
| Present data at regional, national, or international meetings |  |  |  |  |  |  | X | X |
| Write and submit manuscript |  |  |  |  |  |  | X | X |

**EXPECTED APPLICATION OF RESULTS**

Specific Aims 1 and 2 will define the test characteristics for VIA and VILI as well as the parameters for a p16^INK4a^ biochemical assay for use in this population, which would be its first trial among HIV-infected women. This will provide not only a direct comparison, but will help validate the use of either or both low-cost tests in the setting of an HIV-care clinic. An ELISA-based biochemical assay can be developed into a simple test that can could be run immediately without water or electricity requirements, similar to a urine pregnancy, blood glucose or HIV-test. Development of a point-of-care test for a biomarker with high sensitivity and specificity for CIN will exponentially increase access to cervical cancer screening in resource-limited settings.

Specific Aims 3 and 4 will provide preliminary data for a larger trial looking at HR-HPV and HR-HPV-related high-grade CIN as risk factors for HIV-transmission. Aim 4 will provide preliminary data about the levels and time-course of post-treatment HIV-1 genital shedding, providing a better understanding of potential risks of cervical dysplasia treatment among HIV-infected women. These findings will guide pre- and post-procedure counseling to ensure women’s safety and minimize risk of HIV transmission as well as aid in the design of a randomized trial that incorporates HIV genital shedding as a safety outcome to be compared by treatment arms.

**REFERENCES**

1. Ferlay J, Bray Fi, Pisani P, Parkin Dm. Globocan 2002: Cancer Incidence, Mortality and Prevalence Worldwide. IARC Cancerbase No. 5 version 2.0. 2004.

2. Koss Lg. The Papanicolou test for cervical cancer detection: a triumph and a tragedy. JAMA. 1989;261737-43.

3. Holschneider Ch, Ghosh K, Montz Fj. See-and-treat in the management of high-grade squamous intraepithelial lesions of the cervix: a resource utlization analysis. Obstet Gynecol. 1999;94377-85.

4. Massad Ls, Meyer Pm. Predicting compliance with follow-up recommendations after colposcopy among indigent urban women. Obstet Gynecol. 1999;94371-76.

5. Mckee Md, Lurio J, Marantz P, Burton W, Mulvihill M. Barriers to follow-up of abnormal Papanicolou smears in an urban community health center. Arch Fam Med. 1999;8(129-34).

6. Robles Sc, White F, Peruga A. Trends in cervical cancer mortality in the Americas. Bull Pan Am Health Organ. 1996;30290-301.

7. Goldie Sj, Grima D, Kohli M, Wright Tc, Weinstein M, Franco E. A comprehensive natural history model of HPV infection and cervical cancer to estimate the clinical impact of a prophylactic HPV-16/18 vaccine. Int J Cancer. 2003;106(6):896-904.

8. Who/Ico Information Centre on Hpv and Cervical Cancer (Hpv Information Centre). Report on HPV and cervical cancer statistics in Kenya, 2007.

9. Parkin Dm, Ferlay J, Hamdi-Cherif M. Cervix Cancer. Cancer in Africa: epidemiology and prevention. Lyon, France: IARC Press, 2003.

10. Goldie Sj, Gaffikin L, Goldhaber-Fiebert Jd, et al. Cost-effectiveness of cervical cancer screening in five developing countries. N Engl J Med. 2005;353(20):2158-68.

11. Gichangi Pb, Bwayo J, Estambale B, et al. Impact of HIV infection on invasive cervical cancer in Kenyan women. Aids. 2003;17(13):1963-8.

12. Centers for Disease Control and Prevention. 1993 Revised classification system for HIV infection and expanded surveillance case definition for AIDS among adolescents and adults. JAMA. 1993;269729-35.

13. Palefsky J. Biology of HPV in HIV infection. Adv Dent Res. 2006;19(1):99-105.

14. Sun X, Kuhn L, Ellerbrock Tv, Chiasson Ma, Bush Tj, Wright Tc. Human papillomavirus infection in women infected with the human immunodeficiency syndrome. N Engl J Med. 1997;3371343-49.

15. Frisch M, Biggar R, Goedert J. Human papillomavirus-associated cancers in patients with human immunodeficiency virus infection and acquired immunodeficiency syndrome. J Natl Cancer Inst. 2000;921500-10.

16. Cubie H, Seagar Al, Beattie Gj, Monaghan S, Williams Arw. A longitudinal study of HPV detection and cervical pathology in HIV infected women. Sex Transm Infect. 2008;76257-61.

17. Volkow P, Rubi S, Lizano M, Carrillo A, Vilar-Compte D, Garcia-Carranca A. High prevalence of oncogenic human papillomavirus in the genital tract of women with human immunodeficiency virus. Gynecol Oncol. 2001;8227-31.

18. Palefsky Jm. Cervical human papillomavirus infection and cervical intraepithelial neoplasia in women positive for human immunodeficiency virus in the era of highly active antiretroviral therapy. Curr Opin Oncol. 2003;15(5):382-8.

19. Strickler Hd, Burk Rd, Fazzari M, et al. Natural history and possible reactivation of human papillomavirus in human immunodeficiency virus-positive women. J Natl Cancer Inst. 2005;97(8):577-86.

20. Mbu E, Kongnyuy E, Tonye R, Nana P, Leke R. Gynaecological morbidity among HIV positive pregnant women in Cameroon. Reprod Health. 2008;5(3).

21. Bongain A, Rampal A, Durant J, Michiels J-F, Dellamonica P, Gillet J-Y. Cervical intra-epithelial neoplasia in women infected with human immunodeficiency virus. Eur J Obstet Gynecol Reprod Biol. 1996;65195-99.

22. Ellerbrock Tv, Chiasson Ma, Bush Tj, Sun X, Sawo D, Brudney. Incidence of cervical squamous intraepithelial lesions in HIV-infected women. JAMA. 2000;293(1471-76).

23. Six C, Heard I, Bergeron C. Comparative prevalence, incidence and short term prognosis of cervical squamous intraepithelial lesions amongst HIV-positive and HIV-negative women. AIDS. 1998;121047-56.

24. Parham Gp, Sahasrabuddhe Vv, Mwanahamuntu Mh, et al. Prevalence and predictors of squamous intraepithelial lesions of the cervix in HIV-infected women in Lusaka, Zambia. Gynecol Oncol. 2006;1031017-22.

25. Strickler Hd, Burk Rd, Fazzari M. Natural history and possible reactivation of human papillomavirus in human immunideficiency virus-positive women. J Natl Cancer Inst 2005;97577-86.

26. Harris T, Burk Rd, Palefsky Jm, Massad Ls, Bang J, Anastos K. Incidence of cervical squamous intraepithelial lesions associated with HIV serostatus, CD4+ cell counts and human papillomavirus test results. JAMA. 2005;2931471-6.

27. Chirenje Zm, Rusakaniko S, Akino V, Mlingo M. A randomised clinical trial of loop electrosurgical excision procedure (LEEP) versus cryotherapy in the treatment of cervical intraepithelial neoplasia. J Obstet Gynaecol. 2001;21(6):617-21.

28. Fruchter Rg, Maiman M, Sedlis A, Bartley L, Camilien L, Arrastia C. Multiple recurrences of cervical intraepithelial neoplasia in women with human immunodeficiency virus. Obstet Gynecol. 1996;87(3):338-44.

29. Gilles C, Manigart Y, Konopnicki D, Barlow P, Rozenberg S. Management and outcome of cervical intraepithelial neoplasia lesions: a study of matched cases according to HIV status. Gynecol Oncol. 2004;96112-18.

30. Kietpeerakool C, Srisomboom J, Suprasert P, et al. Outcomes of loop electrosurgical excision procedure for cervical neoplasia in human immunodeficiency virus-infected women. Int J Cancer. 2006;161082-88.

31. United Nations Programme on Aids (Unaids)/World Health Organization (Who). AIDS Epidemic update: December 2006. Geneva: UNAIDS/WHO, 2006.

32. United Nations Programme on Aids (Unaids). Report on the Global AIDS Epidemic. 2008.

33. Kenya Aids Indicator Survey. 2008.

34. Blumenthal Pd, Lauterbach M, Sellors Jw, Sankaranarayanan R. Training for cervical cancer prevention programs in low-resource settings: focus on visual inspection with acetic acid and cryotherapy. Int J Gynecol Obstet. 2005;89S30-S37.

35. Denny L, Sankaranarayanan R. Secondary prevention of cervical cancer. Int J Gynecol Obstet. 2006;94(S1):S65-70.

36. Denny L, Kuhn L, De Souza M, Pollack A, Dupree W, Wright Tc. Screen-and-treat approaches for cervical cancer prevention in low-resource settings: a randomized controlled trial. JAMA. 2005;294(17):173-81.

37. Sankaranarayanan R, Nene Bm, Dinshaw K, et al. A cluster randomized trial of visual, cytology and human papillomavirus screening for cancer of the cervix in rural India. Int J Cancer. 2005;116617-23.

38. Sankaranarayanan R, Esmy Po, Rajkumar R, et al. Effect of visual screening on cervical cancer incidence and mortality in Tamil, Nadu, India: a cluster-randomised trial. Lancet. 2007;370398-406.

39. Belinson Jl, Pretorious R, Zhang Wh, Wu Ly, Qiao Yl, Elson P. Cervical cancer screening by simple visual inspection after acetic acid. Obstet Gynecol. 2001;98(3):441-4.

40. De Vuyst H, Claeys P, Njiru S, et al. Comparison of pap smear, visual inspection with acetic acid, human papillomavirus DNA-PCR testing and cervicography. Int J Gynecol Obstet. 2005;89(2):120-26.

41. Denny L, Kuhn L, Pollack A, Wainwright H, Wright Tc. Evaluation of alternative methods of cervical cancer screening in resource poor settings. Cancer 2000;89823-26.

42. Gaffikin L, Mcgrath J, Arbyn M, Blumenthal Pd. Visual inspection with acetic acid as a cervical cancer test: accuracy validated using latent class analysis. BMC Med Res Metholod. 2007;7.

43. Boardman La, Cotter K, Raker C, Cu-Uvin S. Cervical Intraepithelial neoplasia grade 2 or worse in human immunodeficiency virus-infected women with mildly abnormal cervical cytology. Obstet Gynecol. 2008;112(2 Pt 1):238-43.

44. Wright Tc, Ellerbrock Tv, Chiasson Ma, Van Devanter N, Sun X. Cervical intraepithelial neoplasia in women infected with human immunodeficiency virus: prevalence, risk factors, and validity of Papinicolau smears. New York Cervical Disease Study. Obstet Gynecol. 1994;84(4):591-97.

45. Maiman M, Fruchter Rg, Sedlis A, et al. Prevalence, risk factors and accuracy of cytologic screening for cervical intraepithelial neoplasia in women with human immunodeficiency virus. Gynecol Oncol. 1998;68(3):233-39.

46. Branca M, Rossi E, Alderisio M, et al. Performance of cytology and colposcopy in diagnosis of cervical intraepithelial neoplasia (CIN) in HIV-positive and HIV-negative women. Cytopathology. 2001;1284-93.

47. Kitchener H, Nelson L, Adams J, et al. Colposcopy if not necessary to assess the risk to the cervix in HIV-positive women: an international cohort study of cervical pathology in HIV-1 positive women. Int J Cancer. 2007;1212484-91.

48. Fahey Mt, Irwig L, Macaskill P. Meta-analysis of pap test accuracy. Am J Epidemiol. 1995;141680-89.

49. Cuschieri K, Wentzensen N. Human Papillomavirus mRNA and p16 detection as biomarkers for the improved diagnosis of cervical neoplasia. Cancer Epidemiol Biomarkers Prev. 2008;17(10):2536-45.

50. Redman R, Rufforny I, Liu C, Wilkinson E, Massoll N. The utility of p16(Ink4a) in discriminating between cervical intraepithelial neoplasia 1 and nonneoplastic equivocal lesions of the cervix. Arch Pathol Lab Med. 2008;132(5):795-99.

51. Gurrola-Diaz Cm, Suarez-Rincon Ae, Vazquez-Camacho G, et al. P16INK immunohistochemistry improves the reproducibility of the histological diagnosis of cervical intraepithelial neoplasia in cone biopsies. Gynecol Oncol. 2008;111(1):120-24.

52. Wentzensen N, Hampl M, Herkert M, et al. Identification of high-grade cervical dysplasia by the detection of p16INK4a in cell lysates obtained from cervical samples. Cancer Cytopathology. 2006;107(9):2307-13.

53. Balasubramian A, Hughes J, Mao C, et al. Evaluation of an ELISA for p16INKa as a screening test for cervical cancer. Cancer Epidemiol Biomarkers Prev. 2009;18(11):3008-17.

54. Wentzensen N, Zuna Re, Sherman Me, et al. Accuracy of cervical specimens obtained for biomarker studies in women with CIN3. Gynecol Oncol. 2009;115(3):493-96.

55. Kenya Demographic and Health Survey. 2003.

56. Chakraborty H, Sen P, Helms R, Vernazza P, Fiscus S, Eron J. Viral burden in genital secretions determines male-to-female sexual transmission of HIV-1: a probabilistic model. AIDS. 2001;15621-27.

57. Vernazza P, Eron J, Fiscus S, Cohen Ms. Sexual transmission of HIV: infectiousness and prevention. AIDS. 1999;13(2):155-66.

58. Serwadda D, Gray R, Sewankambo N, et al. Human immunodeficiency virus acquisition associated with genital ulcer disease and herpes simplex type 2 infection: a nested case-control study in Rakai, Uganda. J Infect Dis. 2003;188(10):1492-97.

59. Mcclelland Rs, Wang Cc, Mandaliya K, Overbaugh J, Reiner M, Panteleeff Dd. Treatment of cervicitis is associated with decreased cervical shedding of HIV-1. AIDS. 2001;15105-10.

60. Lawn Sd, Subbarao S, Wright Tc, et al. Correlation between human immunodeficiency virus type 1 RNA levels in the female genital tract and immune activation associated with ulceration of the cervix. J Infect Dis. 2000;1811950-56.

61. Legoff J, Weiss Ha, Gresenguet G. Cervicovaginal HIV-1 and herpes simplex virus type 2 shedding during genital ulcer disease episodes. AIDS. 2007;211569-78.

62. Hashemi Fb, Ghassemi M, Faro S, Aroutcheva A, Spear Gt. Induction of human immunodeficiency virus type-1 expression by anaerobes associated with bacterial vaginosis. J Infect Dis. 2000;184924-30.

63. Coleman Js, Hitti J, Bukusi Ea, et al. Infectious correlates of HIV-1 shedding in the female upper and lower genital tracts. AIDS. 2007;21(6):755-59.

64. Kilmarx Ph, Mock Pa, Levine Wc. Effect of *Chlamydia trachomatis* infection on HIV shedding in genital tract secretions. Sex Transm Dis. 2001;28347-48.

65. Mostad S, Kreiss J. Shedding of HIV-1 in the genital tract. AIDS. 1996;101305-15.

66. Ghys Pd, Fransen K, Diallo Mo, Ettiegne-Traore V, Coulibaly Im, Yeboue Km. The associations between cervicovaginal HIV shedding, sexually transmitted diseases and immunosuppression in female sex workers in Abidjan, Cote d'Ivoire. AIDS. 1997;11F85-93.

67. Wang Cc, Mcclelland Rs, Reilly M, Overbaugh J, Emery Sr, Mandaliya K. The effect of treatment of vaginal infections on human immunodeficiency virus type 1. J Infect Dis. 2001;1831017-22.

68. Spinillo A, Zara F, Gardella B, Preti E, Gaia G, Maserati R. Cervical intraepithelial neoplasia and cervicovaginal shedding of human immunodeficiency virus. Obstet Gynecol. 2006;107(2):314-20.

69. Cummins Je, Christensen L, Lennox Jl, et al. Mucosal innate immune factors in the female genital tract are associated with vaginal HIV-1 shedding independent of plasma viral load. AIDS Res Hum Retroviruses. 2006;22(8):788-96.

70. Zara F, Nappi Re, Brerra R, Migliavacca R, Maserati R, Spinillo A. Markers of local immunity in cervico-vaginal secretions of HIV infected women: implications for HIV shedding. Sex Transm Infect. 2004;80108-12.

71. Bollen Ljm, Tjong-a-Hung Sp, Van Der Velden J, et al. Prediction of recurrent and residual cervical dysplasia by human papillomavirus detection among patients with abnormal cytology. Gynecol Oncol. 1999;72199-201.

72. Aerssons A, Claeys P, Beerens E, et al. Prediction of recurrent disease by cytology and HPV testing after treatment of cervical intraepithelial neoplasia. Cytopathology. 2008.

73. Wright Tc, Jr., Subbarao S, Ellerbrock Tv, et al. Human immunodeficiency virus 1 expression in the female genital tract in association with cervical inflammation and ulceration. Am J Obstet Gynecol. 2001;184279-85.

74. World Health Organization. Comprehensive cervical cancer control: a guide to essential practice. Geneva: World Health Organization, 2006.

75. International Agency for Research on Cancer (Iarc). IARC handbooks of cancer prevention volume 10: cervix cancer screening. Lyon, France: IARC Press, 2005.

76. La Ruche G, Ramon R, Mensah-Ado I, et al. Squamous intraepithelial lesions of the cervix, invasive cervical carcinoma, and immunosuppression induced by human immunodeficiency virus in Africa. Cancer. 1998;82(12):2401-8.

77. Personal Communication with Josephine Odoyo Study Coordinator. The Couples Intervention Sudy: a phase 3 randomized placebo-controlled trial of HSV-2 suppression to prevention HIV transmission among HIV discordant couples. unpublished data: Partners in Prevention University of Washington-KEMRI Collaboration, 2008.

78. Huchko Mj, Bukusi Ea, Oyanga A, Cohen Cr. Detection and Treatment of Cervical Dysplasia among Women attending an HIV Care and Treatment Center in Western Kenya Abstract presented at the Annual Meeting of the Infectious Disease Society for Obstetrics and Gynecology. Seattle, Washington, 2008.

79. De Vuyst H, Lillo F, Broutet N, Smith Js. HIV, human papillomavirus and cervical neoplasia and cancer in the era of highly active antiretroviral therapy. Eur J Cancer Prev. 2008;17(6):545-54.

80. Bosch F, De Sanjose S. The epidemiology of human papillomavirus and cervical cancer. Dis Markers. 2007;23(4):213-27.

81. Palefsky Jm. Human papillomavirus-related tumors in HIV. Curr Opin Oncol. 2006;18(5):463-68.

82. Iverson Akn, Atterman J, Gerstoft J, Fugger L, Mullins Ji, Skinhoj P. Longitudinal and cross-sectional studies of HIV-1 RNA and DNA loads in blood and the female genital tract. Europ J Obstet Gynecol Reprod Biol. 2004;117227-34.

83. Carley S, S. D, Jones Sr, Harrison M. Simple nomograms to calculate sample size in diagnostic studies. Emer Med Jour. 2005;22(3):180-81.

84. PASS: Power Analysis and Statistical Software. NCSS, Kayesville, UT. 2008.

85. Ochodo-Opondo E, Huchko Mj, Koech J, Bukusi Ea, Cohen Cr. Cervical cancer screening among HIV-infected women: is cervical biopsy necessary?International AIDS Society Annual Meeting. Cape Town, South Africa, 2009.

86. Spinillo A, Debiaggi M, Zara F, De Santolo A, Polatti F, Filice G. Human Immunodeficiency virus type-1 related nucleic acids and papillomavirus DNA in cervicovaginal secretions of immunodeficiency virus-infected women. Obstet Gynecol. 2001;97999-1004.

87. Hulley Sb, Cummings Sr, Browner Ws, Grady Dg, Newman T. Designing Clinical Research. Philadephia: Lippincott Williams & Wilkins, 2007.

88. John Gc, Haynes S, Mbori-Ngacha D, et al. Comparison of techniques for HIV-1 RNA detection and quantitation in cervicovaginal secretions. J Acquir Immune Defic Syndr. 2001;176170-75.

89. Walker P, Dexeus S, De Palo G, et al. International Terminology of Colposcopy: an updated report from the International Federation for Cervical Pathology and Colposcopy. Obstet Gynecol. 2003;101175-77.

90. Mitchell Mf, Schottenfeld D, Tortolero-Luna G, Cantor Sb, Richards-Kortum R. Colposcopy for the diagnosis of squamous intraepithelial lesions: a meta-analysis. Obstet Gynecol. 1998;91(4):626-31.

91. University of Zimbabwe/Jhpiego Cervical Cancer Project. Visual inspection with acetic acid for cervical cancer. Lancet. 1999;353869-73.

92. Gaffikin L, Mcgrath J, Arbyn M, Blumenthal Pd. Avoiding verification bias in screening test evaluation in resource poor settings: a case study from Zimbabwe. Clin Trials. 2008;5(5):496-503.

93. Arbyn M, Sankaranarayanan R, Muwonge R, et al. Pooled analysis of the accuracy of five cervical cancer screening tests assessed in eleven studies in Africa and India. Int J Cancer. 2008;123153-60.

94. Muwonge R, Walter Sd, Wesley R, et al. Assessing the gain in diagnostic performance when two visual inspection methods are combined for cervical cancer prevention. J Med Screen. 2007;14(3):144-50.

95. Shastri S, Ketayun D, Amin G. Concurrent evaluation of visual, cytological and HPV testing as screening methods for the early detection of cervical neoplasia in Mumbai, India. Bull World Health Organ. 2005;83(3):186-94.

96. Sankaranarayanan R, Basu P, Wesley R, et al. Accuracy of visual screening for cervical neoplasia: results from an IARC multicentre study in India and Africa. Int J Cancer. 2004;110907-13.

97. Megevand E, Denny L, Dehaeck K, Soeters R, Bloch B. Acetic acid visualization of the cervix: an alternative to cytologic screening. Obstet Gynecol. 1996;88383-86.

98. Cecchini S, Bonardi R, A M. Testing cervicography and cervicoscopy as screening tests for cervical cancer. Tumori;7922-25.

99. Singh V, Sehgal A, Parashari A. Early detection of cervical cancer through acetic acid application-an aided visual inspection. Singapore Med J. 2001;42351-54.

100. Rodriguez-Reyes Er, Cerda-Flores Rm, Quinonez-Perez Jm. Acetic acid test: a promising screening test for early detection of cervical cancer. Anal Quant Cytol Histol. 2002;24134-36.

101. Sankaranarayanan R, Rajkumar R, Theresa R, et al. Initial results from a randomized trial of cervical visual screening in rural south India. Int J Cancer. 2004;109461-67.

102. Frisch Le, Milner Fh, Ferris Dg. Naked-eye inspection of the cervix after acetic acid application may improve the predictive value of negative cytologic screening. J Fam Prac. 1994;39457-60.

**BUDGET**

|  |  |  |  |  |  |  |  |
| --- | --- | --- | --- | --- | --- | --- | --- |
| ***Personnel*** | |  |  | *%* | *Salary* | *Benefits* | *Amount* |
| 251 | |  |  |  |  |  |  |
|  | PI: Megan Huchko |  |  | 85% | In-Kind |  |  |
|  | Study Coordinator-KEMRI/FACES |  |  | 100% | $9,600 | $2,021 | $11,621 |
|  | Study Clinician-KEMRI/FACES |  |  | 100% | $6,700 | $1,586 | $8,286 |
|  | Data Analyst/Project Asst-UCSF |  |  | 40% | $50,000 | $5,800 | $25,800 |
|  | Data Entry Clerk-KEMRI/FACES |  |  | 60% | $4,250 | $762 | $3,312 |
| *Sub-total: Personnel* | |  |  |  |  |  | $49,019 |
| ***Non-Personnel Operating Expenses*** | |  |  |  |  |  |  |
|  | |  |  |  |  |  |  |
|  | Genital Viral Load Testing |  |  |  |  |  | $25,110 |
|  | Serum Viral Load Testing |  |  |  |  |  | $3,100 |
|  | STI Testing |  |  |  |  |  | $12,600 |
|  | Pathology |  |  |  |  |  | $4,000 |
|  | Participant Reimbursement |  |  |  |  |  | $3,645 |
|  | Lab and Clinical Supplies |  |  |  |  |  | $10,800 |
|  | Equipment Maintenance |  |  |  |  |  | $600 |
|  | Specimen Shipment |  |  |  |  |  | $2,500 |
|  | Printing |  |  |  |  |  | $800 |
|  | Translation of Consents |  |  |  |  |  | $600 |
|  | Clinician Training (hall rental, meal) | |  |  |  |  | $335 |
|  | Telephone fees: Local and International |  |  |  |  |  | $1,800 |
|  |  |  |  |  |  |  |  |
|  | ***Operating Expenses Subtotal*** | |  |  |  |  | ***$65,098*** |
| **DIRECT COSTS US$** | |  |  |  |  |  | **$114,909** |
| **DIRECT COSTS Kenyan Shillings** | |  |  |  |  |  | **8,618,175**  **Ksh** |

Budget Justification

**PERSONNEL**

**Principal Investigator, Megan Huchko, MD, MPH, (salary support provided by KL2 award)**

As Principal Investigator, Dr. Huchko will be responsible for the overall scientific governance and programmatic leadership of the study. Dr. Huchko will provide oversight of implementation of all study components, including fiscal and administrative management of the study. She will hire and train the study coordinator, lead the development of the study protocol and manual of procedures, oversee submissions to ethical review committees for KEMRI and UCSF, train the study clinicians, and oversee enrollment of study participants and maintenance of good clinical practices. Dr. Huchko will also visit the program site in Kenya at least twice to monitor program progress and to guide program development She will be in regular communication with the scientific collaborators and study coordinator through conference calls, meetings, and email correspondence.

**Study Coordinator (FACES), TBD (12 calendar months)**

An on-site study coordinator will be essential for the smooth functioning of the research project. Responsibilities include assisting the PI with implementation of the protocol, coordinating communication with the KEMRI ethical review committee, ensuring that the recruitment and follow-up goals of the study are met, providing guidance in study-related procedures and good clinical practice to study clinicians, and providing general day-to-day study administration. He/she will be in regular communication with the PI via phone and email. He/she will have training s a nurse and significant experience in research.

**Study Clinician (FACES), TBD (12 calendar months)**

The study clinician will assist the study coordinator in carrying our the clinical tasks as part of the study visits, including the study-related colposcopy and specimen collection. This person will under good clinical practices and study-specific training and will assist the study coordinator and PI in day-to-day management and continued implementation of the study.

**Data Analyst/Project Assistant (UCSF), TBD (4.8 calendar months)**

The Data Analyst/Project Assistant will assist the PI in submission of ethical review committee documents throughout the duration of study, development of training materials and the study manual of operations, creation of the participant database and SOPs for the Kenyan-based data clerk and preparation of study forms and documents. In collaboration with Dr. von Knebel Doeberitz, he/she will be responsible for cleaning, managing, and analyzing data collected from study sites for analysis and reporting. Fringe benefits for UCSF personnel are calculated at 29%.

**Data Entry Clerk (FACES), TBD (6.6 calendar months)**

The data entry clerk will with the Data Analyst/Project Assistant to create the study database and will be responsible for verifying that data is entered with accuracy, completeness and consistency. He/she will also maintain orderliness and confidentiality of participants’ paper files.

KEMRI contract personnel are paid a 15% gratuity at the end of their contract period and benefits include medical insurance cover at $490 per person, based on percent effort, Worker’s Injury Benefit Insurance at $59 per person and the NSSF contribution of $2.65 per calendar month of employment.

**OTHER**

**Laboratory Testing:**

**Cervico-vaginal HIV-1 Viral Loads ($25,110)**

Viral load testing will be performed with the assistance of Teri Liegler through the ARI-UCSF LCV/CFAR Virology Core Lab. There will be a total of 100 participants: 50 women with no evidence of cervical dysplasia who have a single cervico-vaginal viral load and 50 women with cervical dysplasia who have one baseline visit (100). Thirty women will be part of the longitudinal study and seven follow-up visits (210). The tests are $81 each, including the strips and collection containers. Storage will be provided in the freezers of the research laboratory in the FACES clinic. Total: 310*81=$25,110.

**Serum Viral Load Testing ($3,100)**

Viral load testing is available through a clinical and research facility affiliated with the University of Nairobi and University of Washington in Nairobi at $31.00 per test. All 100 women in specific aims 1 and 2 will have baseline serum viral load testing. Total: 100*31=$3,100.

**STI Testing ($12,600)**

NAAT Testing for sexually transmitted infections will be carried out in a local facility at approximately $7 per test. Total: 1800*6=$12,600.

**Pathology Reading ($4,000)**

Pathology specimens will be read by a trained pathologist at KEMRI, with whom there is an established relationship for FACES and the cervical cancer screening program. From our current rate of positive colposcopies and detection of CIN2/3, we have estimated that approximately 500 specimens will be read at $8 per specimen for this study. The UCSF pathologist has agreed to provide a second read of the prepared specimens at no extra fee. Total: 500*8=$4,000.

**Participant Reimbursement ($1,150)**

Participants in the cross-sectional/case control study (specific aims 1, 2 and 3) will be reimbursed 100Ksh/$1.50 in the form of a meal voucher for their participation (1800*1.50=$2700). Participants in the longitudinal study (specific aim 4) will be reimbursed 300Ksh ($4.50) per visit for transportation (210 X 4.50= $945). Total: 2700+ 945= 1150

**Lab and Clinical Supplies ($10,800)**

Lab and clinical supplies include distilled water for the autoclave, specula and dispensibles for the individual colposcopic examinations, including swabs, acetic acid and Lugol’s iodine, light bulbs, labels, formalin and specimen containers. These have been estimated at $6 per participant. 1,800*6=$10,800.

**Equipment Maintenance ($600)**

The study will use the existing colposcope already in place as part of the cervical cancer screening study. We request $600 for cleaning and maintenance of the colposcope.

**Shipping ($2,500)**

We request $2,100 for shipping specimens to the laboratory at the German Cancer Research Center in Heidelberg, Germany for processing and evaluation. The additional $400 will be used to ship pathology specimens to the UCSF pathologist for the additional review and quality assurance.

**Photocopying/Printing ($550)**

This includes reproducing consent and data collection forms, training materials and participant information sheets.

**Translation Services ($1000)**

We have estimated for translation of two 5-page consent forms into Dhluo and Kswahili, at a rate of $50 per page.

**Study Specific Training ($350)**

The study procedures will be carried out by FACES staff members, who will undergo training in good clinical practices and study-specific procedures. This includes the rental of a conference room and lunch to be provided for clinical officers for two-days of training.

**Telephone ($1800)**

We have budgeted $1800 to cover local and international telephone expenses for this study.

**APPENDICES**

A. Information and Consent form, Safety and Validity, English V2: 9 April 2010

B. Information and Consent form, Safety and Validity Participants, Dholuo V2: 9 April 2010

C. Information and Consent form, Safety and Validity Participants, Kiswahili V2: 9 April 2010

D. Information and Consent form, post-LEEP Shedding, English V1: 14 March 2010

E. Information and Consent form, post-LEEP Shedding, Dholuo V1: 14 March 2010

F. Information and Consent form, post-LEEP Shedding, Kiswahili V1: 14 March 2010

G. Safety and Validity Study Information Sheet, V2: 9 April 2010

H. Post-LEEP Shedding Information Sheet, V1: 14 March 2010

I. Study Flowsheet, V2: 9 April 2010

J. Colposcopy Data Collection Form, V1: 14 March 2010

K. Follow-up Interview Form, V1: 14 March 2010

L. Information and Consent form, p16^INK4a^ follow-up study, 8 October 2011
